# Diversity in the Common Fold: Structural Insights into Class D β-Lactamases from Gram-Negative Pathogens

**DOI:** 10.3390/pathogens14080761

**Published:** 2025-08-01

**Authors:** Clyde A. Smith, Anastasiya Stasyuk

**Affiliations:** 1 Stanford Synchrotron Radiation Lightsource, SLAC National Accelerator Laboratory, Menlo Park, CA 94025, USA; 2 Department of Chemistry, Stanford University, Stanford, CA 94303, USA; 3 University of California Davis Medical Center, Sacramento, CA 95817, USA

**Keywords:** gram-negative, antibiotic resistance, β-lactamase, carbapenemase, structural dynamics

## Abstract

Class D β-lactamases (DBLs) represent a major threat to antibiotic efficacy by hydrolyzing β-lactam drugs, including last-resort carbapenems, thereby driving antimicrobial resistance in Gram-negative bacteria. The enzymes share a structurally conserved two-domain α/β architecture with seven active-site motifs and three flexible extended loops (the P-loop, Ω-loop, and newly designated B-loop) that surround the active site. While each of these loops is known to influence enzyme function, their coordinated roles have not been fully elucidated. To investigate the significance of their interplay, we compared the sequences and crystal structures of 40 DBLs from clinically relevant Gram-negative pathogens and performed molecular dynamics simulations on selected representatives. Combined structural and dynamical analyses revealed a strong correlation between B-loop architecture and carbapenemase activity in the pathogens *Klebsiella* and *Acinetobacter*, particularly regarding loop length and spatial organization. These findings emphasize the B-loop’s critical contribution, in concert with the P- and Ω-loops, in tuning active site versatility, substrate recognition, catalytic activity, and structural stability. A deeper understanding of how these motifs and loops govern DBL function may inform the development of novel antibiotics and inhibitors targeting this class of enzymes.

## 1. Introduction

**Figure 1:**
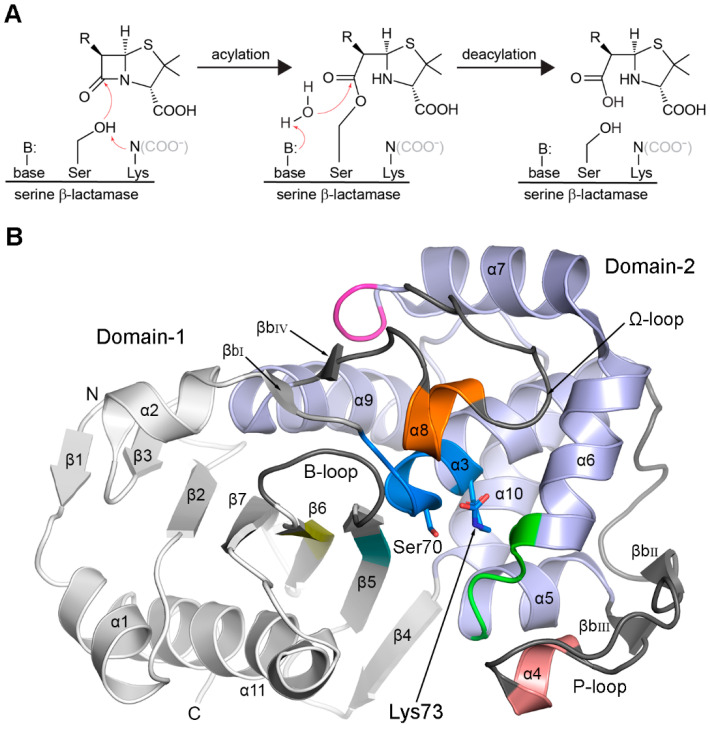
Mechanism and structure of a serine β-lactamase. (**A**) The serine β-lactamase two-step enzymatic mechanism. In all three classes, the serine is activated by donating a proton to the catalytic lysine. In class D enzymes, this lysine is post-translationally modified by carboxylation (indicated by the COO- in gray). During deacylation, the general base is a glutamate in class A enzymes, tyrosine in class C, and the same carboxylated lysine in class D. (**B**) Ribbon representation of the two structural domains (Domain-1, gray; Domain-2, light blue) of the class D β-lactamase OXA-48. The secondary structure annotation (helices, strands and isolated β-bridges) is indicated. Sequence motifs specific for the class D enzymes are colored as follows: *motif-1* (blue), *motif-2* (pink), *motif-3* (green), *motif-4* (magenta), *motif-5* (orange), *motif-6* (cyan) and *motif-7* (yellow). The three variable loops (P-loop, Ω-loop and the B-loop) are colored black and labeled. The locations of the catalytic serine and lysine residues are indicated.

The β-lactamases, bacterial enzymes that hydrolyze β-lactam antibiotics (penicillins, cephalosporins, monolactams, and carbapenems), are implicated in the resistance of bacteria to these therapeutic compounds [1,2,3,4,5]. There are over 11,500 β-lactamase enzymes known [6] (Figure S1), and these can be divided into four molecular classes (A, B, C, and D) based on amino-acid sequence similarity [7,8]. Enzymes from classes A, C, and D are serine β-lactamases, and those in class B are zinc-dependent metalloenzymes [5,9,10]. The class A, C, and D enzymes have a similar two-step mechanism involving (1) the formation of a covalent acyl-enzyme intermediate following an attack on the β-lactam ring by the active site serine and (2) the subsequent deacylation of the intermediate involving cleavage of the acyl bond facilitated by an activated water molecule (the deacylating water) (Figure 1A). In the deacylation step of the mechanism, we begin to see specific differences between the three enzyme classes in the way in which the deacylating water molecule becomes activated. In class A enzymes, a conserved glutamate residue acts as a general base to abstract a proton from the deacylating water molecule, and in class C enzymes, a conserved tyrosine is used. In class D β-lactamases (DBLs), a universally-conserved lysine residue that is one turn of helix away from the catalytic serine (Figure 1B) is post-translationally modified by carboxylation in order for it to subsequently serve as the base to deprotonate the deacylating water [11]. Decarboxylation of this lysine, either under low pH conditions or through the binding of compounds that trigger the removal of the CO_2_, effectively inhibits the DBLs [12,13,14,15].

The serine β-lactamases share two highly conserved sequence motifs, SxxK containing the catalytic serine and lysine residues (the latter modified by carboxylation), and the K(S/T)G motif adjacent to the active site (Figure 1B). A third motif, Sxx on a short helix–helix loop near the active site, is topologically common to all three classes (as the SDN motif in class A, the YA(S)N motif in class C, and the SAV motif in class D) [16]. The tyrosine residue in the YA(S)N motif in class C enzymes is the general base involved in deacylation. It should be noted that early reports [17] erroneously equated another highly conserved class D motif (YGN) as being equivalent to the SDN and YA(S)N motifs, but these analyses were performed using only sequence comparisons, since structural information on the DBLs was unknown in the late 1990s. Once the first DBL structures became available [18,19], it was evident that the highly-conserved SAV motif is the structural equivalent of the SDN and YA(S)N in the Class A and C enzymes [16]. The aforementioned YGN motif is, in fact, on the opposite side of the enzyme (Figure 1B) and can now be considered a fourth DBL fingerprint motif.

The earliest discovered DBLs, classified as narrow-spectrum β-lactamases (NSBLs) [20], were all from Gram-negative *Pseudomonas*. Since they hydrolyze oxacillin more efficiently than benzylpenicillin [9], they were given the name OXA (for example, OXA-1, OXA-2, OXA-5, and OXA-10). Variants of these enzymes, primarily derived from OXA-2 and OXA-10 as point mutations of the parental enzyme, have since been identified with a broader spectrum of activity for β-lactams, and these have been classified as extended-spectrum β-lactamases (ESBLs) capable of hydrolyzing third-generation cephalosporins and aztreonam [20]. More disturbingly, a carbapenem-hydrolyzing ability has evolved, first observed with the OXA-23 [21] and OXA-24 [22] enzymes found in *Acinetobacter baumanni* isolates, and the OXA-48 enzyme from *Klebsiella pneumoniae* [23]. Collectively, the carbapenem-hydrolyzing class D β-lactamases are known as CHDLs [24]. There are now almost 1430 DBLs known [6], the majority from Gram-negative bacteria, although Gram-positive enzymes have recently been reported [25,26,27]. Strong familial relationships amongst the Gram-negative DBLs (GnDBLs) can be readily detected at the sequence level (Table S1). There are 50 OXA families (a family is defined as a group of two or more sequences with greater than 90% identity), three additional families not annotated as OXA enzymes, and 38 orphan enzymes with no familial relationships. There are currently 22 families of OXA enzymes that have been annotated as CHDLs (Table S1), numbering over 920 enzymes in total. The majority of these carbapenemases have been isolated from *Acinetobacter* sp., which have rendered most strains of this pathogen highly resistant to these last resort antibiotics [28].

To date, crystal structures have been determined for 40 unique GnDBLs, representing 13 different families and five orphans (Table S2). A standardized secondary structure annotation has recently been reported for these enzymes [29,30]. Although these enzymes share a conserved overall fold, comparative structural analyses recently revealed divergence in two external loops, the P-loop and the Ω-loop, which flank the active site [29]. These two loops, together with a third extended loop near the opening of the active site, have been implicated in substrate recognition and catalytic activity; however, the structural relationships among them and how their dynamic interplay may modulate enzymatic function are not fully understood. Here, we present a detailed comparative analysis aimed at elucidating how variability in loop architecture contributes to functional diversification among GnDBLs.

## 2. Materials and Methods

### 2.1. Sequence and Structural Analysis

Amino acid sequences for GnDBLs were identified from the β-lactamase Database (BLDB) [6] and downloaded from GenBank (www.ncbi.nlm.nih.gov/genbank/, accessed on 1 April 2025). Additional OXA enzymes not listed in the BLDB were also found in GenBank and downloaded. A multiple sequence alignment was performed using Clustal Omega v.1.2.6 [31] (www.ebi.ac.uk/jdispatcher/msa/clustalo, accessed on 7 April 2025), and the resulting alignment was visualized with JALVIEW [32] to analyze the sequence variation in the seven DBL motifs. A multiple sequence alignment of the 40 GnDBLs whose structures are in the Protein Data Bank (PDB) was also generated with Clustal Omega and manually adjusted based upon a superposition of all the structures.

The structures were superimposed using the secondary structure matching (SSM) algorithm [33], as implemented in COOT [34]. The OXA-48 structure (PDB code 3HBR) was used as the reference molecule for the superposition. All structural figures were generated with PyMOL (v2.6.0) from Schrödinger (www.pymol.org).

### 2.2. Molecular Dynamics Simulations

Molecular dynamics (MD) simulations were performed in triplicate for 250 ns on six GnDBLs: OXA-1, OXA-10, OXA-48, OXA-143, OXA-225, and OXA-427, chosen for the diversity seen in their P-loop, Ω-loop, and B-loop structures. Simulations were carried out using Desmond [35] as implemented in the Schrödinger 2019-2 software suite. System preparation was conducted in Maestro (Schrödinger) using the OPLS3e force field [36]. Solvation was achieved with the pre-defined TIP3P water model [37]. The net charge of each system was neutralized with either Na^+^ or Cl^−^ counterions, and 0.15 M NaCl was added to mimic physiological ionic strength. Following energy minimization, production runs were executed for 250 ns at 300 K and 1 atm. Temperature and pressure were controlled using the Nosé–Hoover chain thermostat and the Martyna–Tuckerman–Klein barostat, respectively, with a pressure coupling constant of 2.0 ps [38]. Long-range nonbonded interactions were computed using an r-RESPA multiple time-step integrator. Simulation trajectories were saved every 10 ps for subsequent analysis. All simulations were performed on the SHERLOCK 3.0 high-performance computing cluster at Stanford University.

## 3. Results and Discussion

### 3.1. The Common DBL Structural Fold

The DBLs all have the same common three-dimensional architecture comprising eleven helices (α1–α11) and seven strands (β1–β7) folded as two structural domains (Figure 1B). Domain-1 is composed of a mostly antiparallel β-sheet with two flanking helices, and Domain-2 is a globular α-helical domain [18,19] decorated with two extended loops. The Domain-1 central β-sheet of five long strands (β2, β3, β5, β6, and β7) is markedly curved, its convex side abutting the helical Domain-2, with two short strands at either end (β1 and β4) (Figure 1B). The two terminal helices (α1 and α11) are nestled against the concave face of the β-sheet. The active site is located at the junction of the two structural domains, with the catalytic serine and lysine residues at the N-terminus of helix α3 (Figure 1B).

While the majority of the secondary structure elements are joined by short turns, there are three long extended loops that surround the active site and play integral roles in enzyme function. The first loop, comprising 27–30 residues between helices α3 and α5, is known as the P-loop [39,40]. This loop has a P-shaped structure with the N- and C-terminal ends held together by two antiparallel β-bridges (βb_II_ and βb_III_), and is unstructured except for a short 3_10_ helix (designated α4 in the standard numbering scheme [29,30]) at the tip of the loop. The second extended loop, designated the Ω-loop, connects helices α7 and α9 and is mostly unstructured, except for a 3_10_ helix (α8), which sits adjacent to the active site, and a β-bridge (βb_IV_) is just prior to helix α9 (Figure 1B). The third loop connects strands β5 and β6 in Domain-1 and sits near the opening of the active site, opposite the P-loop. It has previously been referred to simply as the β5–β6 loop, or loop-1 in a study of the Gram-positive DBLs from *Bacillus* sp. [41]; however, rather than apply a numerical descriptor that may create future confusion, for simplicity, this third loop is hereinafter designated the B-loop.

### 3.2. Class D Fingerprint Sequence Motifs

**Figure 2:**
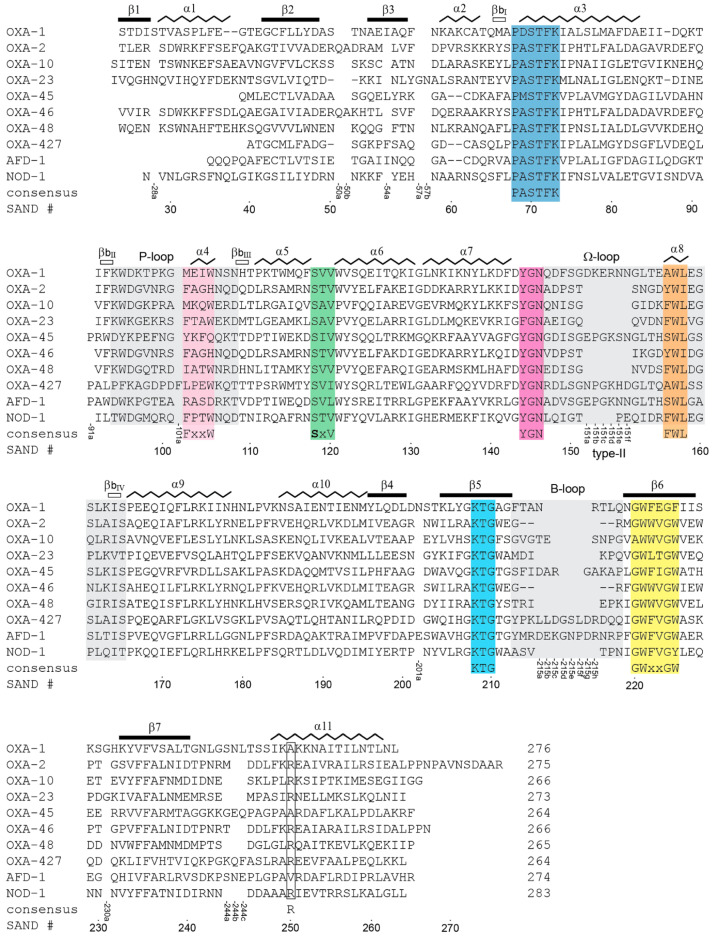
Multiple structure-based sequence alignment of selected GnDBLs. The secondary structure annotation is given above each alignment block. The P-loop, Ω-loop and B-loop are highlighted in gray. The seven sequence motifs are colored as in Figure 1B, and their consensus sequence is given below each alignment block, along with the SAND [29,30] numbering scheme (based on OXA-48 residue numbering). In this scheme any residues missing relative to OXA-48 are skipped in numbering and shown as a dash (-) to maintain alignment. Where there is an insertion in an aligned sequence relative to OXA-48, lowercase letters are added to the residue number that just precedes the insertion, and these numbers are shown vertically at the bottom of each alignment block.

Seven specific sequence motifs (Table 1) can be identified from an alignment of all known GnDBLs; (1) the catalytic motif PASTFK (which includes the universally-conserved serine β-lactamase SxxK motif) at the N-terminus of helix α3, (2) FxxW within the P-loop, (3) SAV in the α5-α6 loop, adjacent to the active site, (4) YGN at the beginning of the Ω-loop, (5) FWL within helix α8 in the Ω-loop, (6) KTG on strand β5, adjacent to the active site and directly opposite the SAV motif, and (7) GWxxGW in the strand β6. The locations of the seven DBL motifs are shown in Figure 1B and are indicated in sequence alignments of selected GnDBLs (Figure 2) and all 40 known structures (Figure S2). Not surprisingly, the sequence motifs are invariably linked to the activity of the enzymes. In the following analysis, the standardized SAND numbering scheme [29,30] will be used, and residue names at particular locations will generally relate to the amino acid with the highest percentage identity (Table 1).

#### 3.2.1. Motif-1 (PASTFK; Residues 68–73)

**Figure 3:**
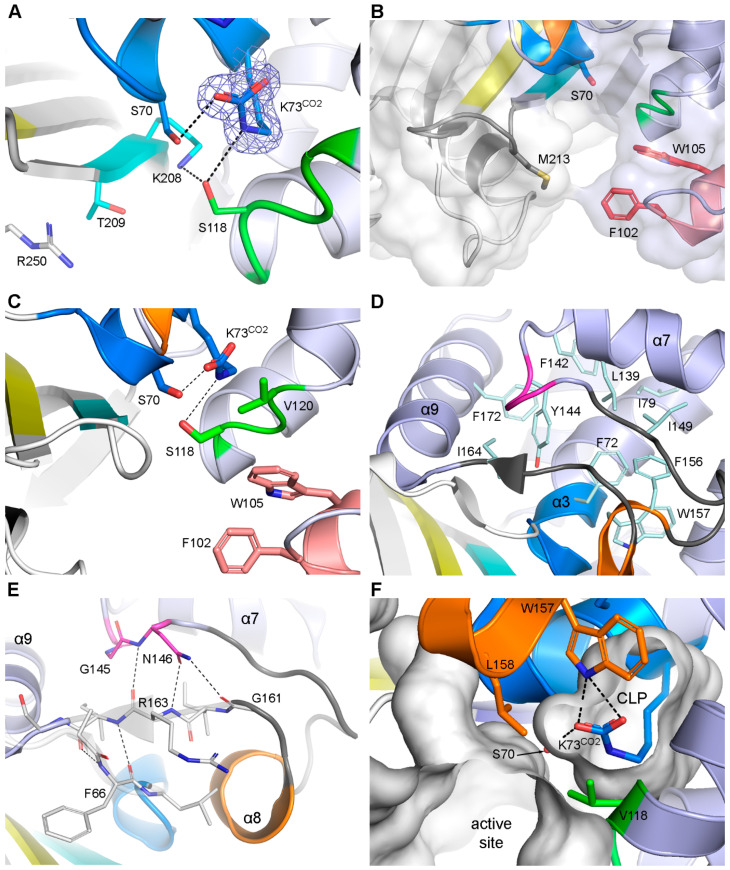
DBL motifs 1–6. (**A**) The active site of OXA-14 (7L5R) showing *motif-1* (blue) at the N-terminus of helix α3 harbors the Ser70 and Lys73^CO2^ catalytic residues. The Lys73^CO2^ side chain (shown in blue mesh representing 2*F_o_*-*F_c_* electron density contoured at 1 σ) interacts with Ser70 and Ser118 from *motif-3* (green), which, in turn, is hydrogen-bonded to Lys208 from *motif-6* (cyan). (**B**) Surface representation of the hydrophobic bridge in OXA-23 (4JF6) formed between Phe102 from *motif-2* and Met213 from the C-terminus of strand β5. (**C**) The active site of OXA-23 showing the proximity of *motif-2* (pink) and *motif-3* (green) from neighboring loops. (**D**) The hydrophobic core buries the aromatic and hydrophobic side chains of ten residues (pale cyan sticks) to stabilize the structure of Domain-2. (**E**) *Motif-4* (magenta) and *motif-5* (orange), the latter part of the Ω-loop (gray). The C-terminal end of the Ω-loop is anchored to the loop at the N-terminus of *motif-1* (blue) by a pair of hydrogen bonds between two isolated β-bridges (βb_I_ and βb_IV_) (thin gray sticks and semi-transparent ribbons). The conserved asparagine of *motif-4* is also hydrogen-bonded to βb_IV_. (**F**) Formation of the catalytic lysine pocket (CLP) and sequestration of Lys73^CO2^ by a pair of hydrophobic residues (Val118 and Leu158) from *motif-3* and *motif-5*, respectively. The Trp157 side chain highly conserved in *motif-5*, is directed into the CLP and stabilizes the orientation of Lys73.

This motif contains two catalytic residues, Ser70 and the carboxylated Lys73 (hereinafter Lys73^CO2^), both universally conserved in all known DBLs from both Gram-negative and Gram-positive bacteria. The leading proline and the threonine at position four are also highly conserved (>99%), while the alanine and phenylalanine are 95.3% and 93% conserved, respectively (Table 1). There is typically a hydrogen bond between one of the carboxylate oxygen atoms of Lys73^CO2^ and the Oγ of Ser70 in the apo form of the enzyme (Figure 3A), and occasionally a second hydrogen bond between the Lys73^CO2^ Nζ atom and the side chain of Ser118 in *motif-2* on the nearby α5-α6 loop. The negatively-charged carboxylate moiety activates Ser70 by abstracting a proton [10], and this is followed by attack by the serine nucleophile on the carbonyl carbon of the β-lactam ring to form a transient tetrahedral intermediate. A shuttle involving the Lys73^CO2^ and Ser118 transfers a proton onto the β-lactam nitrogen [10,15], cleaving the β-lactam ring to form a moderately stable acyl-enzyme intermediate, and leaving the Lys73^CO2^ with a negative change once again (Figure 1A). As noted earlier, in the second step of the reaction, the Lys73^CO2^ activates a water molecule that subsequently breaks the acyl bond to generate inactive penicillinoic acid.

#### 3.2.2. Motif-2 (FxxW; Residues 102–105)

This motif is located at the tip of the P-loop, forming the outer edge of one side of the active site (Figure 1B) and encompasses the 3_10_ helix α4. Although the first residue at position 102 shows a high degree of variability (Table 1), the class D carbapenemases (CHDLs) from *A. baumannii* (OXA-23, OXA-24, OXA-51, OXA-58, and OXA-143) invariably have Phe102 or Tyr102 in this location. This residue extends across the active site cleft and makes a hydrophobic interaction with the highly conserved Met213 or Trp213 at the C-terminus of strand β5, producing a structural feature known as the hydrophobic bridge (Figure 3B). It was shown in OXA-24 that the presence of this bridge favored the formation of the Δ^2^ tautomer of carbapenem substrates over the Δ^1^ tautomers [42,43], with the former being more efficiently hydrolyzed. It was later demonstrated in the case of OXA-23 that although the Δ^1^S tautomer was favored in this enzyme it was still susceptible to hydrolysis despite the presence of the bridge [44], and further mutagenesis studies suggested that it was the stabilizing interactions between the bridge residues and the carbapenem tail that were more important than the identity of the tautomer, with respect to hydrolytic efficiency [45]. The side chain of Trp105 is also generally directed into the enzyme active site (Figure 3B), where it can potentially interact with substrates and inhibitors [44,46], in addition to other residues lining the active site.

#### 3.2.3. Motif-3 (SAV; Residues 118–120)

*Motif-3* is located on the short loop between helices α5 and α6, adjacent to *motif-2* but much deeper into the active site (Figure 1B and Figure 3C). As noted above, the universally conserved Ser118 can form hydrogen bonds with Nζ of the catalytic lysine and the β-lactam nitrogen of bound substrates [43,47], and constitutes part of the proton shuttle that leads to the cleavage of the β-lactam ring. The conserved residue at the third position of *motif-3* (predominantly Val120; Table 1) is involved in hydrophobic interactions with the side chain of Trp105 from *motif-2* (Figure 3C), which serves to anchor the P-loop to the α5-α6 loop such that both residues then participate in the formation of a hydrophobic side wall of the active site. More importantly, Val120 also interacts with a conserved hydrophobic residue from *motif-5* to sequester Lys73^CO2^ and regulate water access, which will be described below.

#### 3.2.4. Motif-4 (YGN; Residues 144–146)

At first glance, this motif seems to be in an odd position in the three-dimensional structure of the class D enzymes to be so universally conserved, although closer inspection shows that it plays a crucial role in the structural stability of key residues in the enzyme active site. The side chain of the aromatic residue in the first position (either Tyr144 or Phe144) projects into a highly hydrophobic internal core formed by helices α3, α7, and α9, and the Ω-loop (Figure 3D), and makes an edge-to-face π-π stacking interaction with Phe172 (a highly conserved residue in helix α9) and a face-to-edge π-π interaction with Phe72 in *motif-1*. This internal pocket, further enhanced by additional conserved hydrophobic residues, forms the rigid core of Domain-2 and holds helix α3, *motif-1*, and the Ω-loop firmly in position. The conserved Gly145 allows the main chain to form a tight type-I β-turn, which brings Asn146 into a position where it makes three hydrogen-bonding contacts with residues 161 and 163 from the Ω-loop (Figure 3E). In this way, the C-terminus of the surface helix α7 is locked in place against the Ω-loop.

#### 3.2.5. Motif-5 (FWL; Residues 156–158)

*Motif-5* is situated approximately 10–15 residues further downstream of *motif-4* and is generally recognized as a short single-turn 3_10_ helix (α8) in the Ω-loop. The Phe156 side chain (~86% conserved) also projects into the hydrophobic core and is involved in an edge-to-face π-π interaction with Phe72, which serves to rigidify helix α8 and anchor it to helix α3. Several OXA families, including the OXA-1 and OXA-12 families from *Aeromonas*, the OXA-22 family from *Ralstonia*, and the OXA-42 and OXA-114 families from *Burkholderia*, have an alanine or serine at position 156. The hydrophobic core is maintained in these enzymes by sequence differences at position 149 in OXA-1, where there is a phenylalanine instead of the small hydrophobic residue in most other enzymes, and in the OXA-42 family (represented in the PDB by OXA-D84) at position 135 on helix α7, where there is also a phenylalanine (Figure S2). The Trp157 side chain nestles alongside the α3 helix, forming one wall of a small internal cavity, hereinafter referred to as the catalytic lysine pocket (CLP), that houses the Lys73^CO2^. Hydrogen-bonding interactions between the Nε2 atom of Trp157 and the oxygen atoms of the modified lysine orient the carboxylate moiety optimally for interaction with Ser70 and Ser118.

The side chain of Leu158 interacts with Val120 from *motif-3* to form a hydrophobic cap (Figure 3F) that separates the CLP from the active site and external milieu [14,44,48]. This structural feature effectively sequesters the Lys73^CO2^, shielding it from solvent exposure and thereby preserving its integrity prior to the deacylation step of catalysis. In some substrate and inhibitor complexes of DBLs, conformational changes in Val120 and Leu158 have been observed, which effectively open a hole or channel in the hydrophobic cap and allow water molecules to enter a region between Lys73^CO2^ and the acyl bond of the intermediate [14,15,44,46,48]. This region is generally referred to as the deacylating water pocket (DWP), and it is expected that a water molecule within this pocket would be positioned appropriately for activation (via proton removal by Lys73^CO2^) and would subsequently attack the acyl bond and lead to deacylation.

The importance of the tightly-packed hydrophobic core in Domain-2, comprising interlocking aromatic and hydrophobic residues, is underscored by the reported structures of OXA-935 from *Pseudomonas aeruginosa* [49], a variant of OXA-10 harboring two point mutations in the Ω-loop, F156S and G160D. Compared to OXA-14, another OXA-10-like enzyme with only the G160D mutation, the activity of OXA-935 is severely impaired. This enzyme was crystallized in two forms, monoclinic (7L5V) and orthorhombic (7N1M), and a high degree of conformational disorder in the Ω-loop exists in the two crystal forms. The missing Phe156 ring greatly destabilizes the loop such that helix α8 is either partially (in 7L5V) or completely unfolded (in 7N1M), and *motif-5* extends away from the enzyme. This results in the opening of the CLP to the external milieu such that the catalytic lysine is exposed to solvent, so that even at pH 8.3 (where the enzyme was crystallized), Lys73 is fully decarboxylated and would be unable to activate a deacylating water molecule. This structural disruption provides a mechanistic explanation for the substantially reduced activity of OXA-935 relative to both OXA-10 and OXA-14 [49], and demonstrates the critical role that the Ω-loop and *motif*-5 play in enzyme activity.

#### 3.2.6. Motif-6 (K(S/T)G; Residues 208–210)

The second of the universal serine β-lactamase motifs is located on strand β5, which forms the side of the active site opposite the P-loop (*motif 2*) and the α5-α6 loop (*motif-3*). The side chain of Lys208 bridges both sides of the active site cleft through a hydrogen-bonding interaction with Ser118, which also serves to anchor the serine in the correct orientation to act as a proton shuttle during acylation. The conserved hydroxyl residue in the second position of *motif-6* (Ser209/Thr209) projects into the active site cleft and is generally involved in anchoring the carboxylate moiety of bound substrate and inhibitor molecules [15,43,44,46,47,50,51,52]. Some enzymes, most notably those from Gram-positive organisms, have a second hydroxyl residue (Thr211), following the conserved Gly210, and this threonine has also been implicated in substrate-binding [53]. However, the most common residue (>67% in all known class D enzymes) at position 211 is tryptophan (Table 1), and when this residue is present, it acts as a hydrophobic side wall of the active site [54].

#### 3.2.7. Motif-7 (GWx(T/V)GW; Residues 220–225)

Located in strand β10, *motif-7* seems to play a structural role similar to *motif-4*, with the side chain of Trp221 providing stability to the N-terminus of helix α3. Trp225 is in a second, smaller hydrophobic core, sandwiched between Phe234 on strand β11 and residues Leu173, Leu176, and Tyr177 on helix α9, lending stability to the β-sheet domain and the interface between the two domains.

### 3.3. The P-Loop

**Figure 4:**
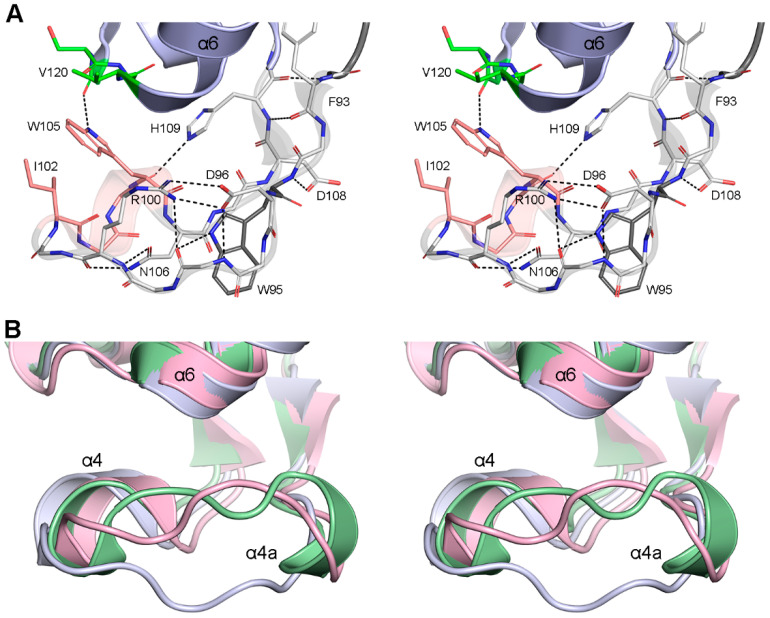
The DBL P-loop. (**A**) Stereoview of the P-loop from OXA-48 (thin gray sticks and semi-transparent ribbons) showing the interactions that hold the loop together and also anchor it to *motif-3* (green) and helix α6. Trp95, which serves as a hydrophobic core around which the rest of the P-loop folds, is colored darker gray. (**B**) Stereoview of the type-I P-loop from OXA-48 (light blue) and the type-II loops from OXA-45 (green) and OXA-427 (pink).

This loop is common to all DBLs whose structures are known, both from Gram-negative and Gram-positive bacteria. The P-loop bends around the side of the α-helical Domain-2 (Figure 1B and Figure 4A), and the tip of the loop (containing *motif-2* and *helix α4*) forms part of the side wall of the active site. The aromatic side chain of position 95 (79% Trp and 18% Tyr in the GnDBLs) creates a hydrophobic core, around which the loop folds, and in most cases, its overall shape is maintained by a salt bridge between the side chains of either an aspartate/arginine pair, or a lysine/aspartate pair at positions 96 and 100, respectively. In addition, the side chain of the residue at position 106 (generally asparagine or glutamate, and occasionally glutamine, lysine, or arginine) reaches across the loop to form two hydrogen bonds with the main chain of either residue 100 or 101 (Figure 4A). In the majority of the GnDBL structures, hydrogen-bonding and hydrophobic interactions between residues on helices α4 and α5 serve to hold the loop against the body of Domain-2. As noted above, for those enzymes that have tryptophan in the fourth position of FxxW *motif-2* (which is the majority of GnDBLs (Figure S2) and >96% of all DBLs), there is a stabilizing hydrophobic interaction between the Trp105 side chain and Val120 (Figure 3C). Moreover, there is typically a hydrogen-bonding interaction between the Nε2 of the tryptophan and the carbonyl oxygen of residue 117, which serves to maintain the Trp105 side chain in the *m0* rotameric conformation, such that it stacks against the invariably hydrophobic residue in the first position of *motif-2* (Table 1 and Figure 4A) and together they project into the active site. Several enzymes, including OXA-1, OXA-2, NOD-1, and STD-1, have a tryptophan at position 121, which is hydrogen-bonded through its Nε2 atom to the carbonyl oxygen of residue 105, further anchoring the loop against helix α6.

Superposition of all 40 unique GnDBLs (Figure S3) reveals that while the two β-bridges (βbII and βbIII) that constrain the ends of the P-loop are topologically conserved across all enzymes, the loop itself exhibits some structural variability. Two distinct families of loops can be identified: a type-I loop, exemplified by the loop in OXA-48 (Figure 4A), is present in 34 of the GnDBL structures, and a type-II P-loop is present in AFD-1, ATD-1, LoxA9, OXA-45, OXA-D84, and OXA-427 (Table S2). Although the β-bridges anchoring the two ends are isostructural in both loop types, and the 3_10_ helix α4 projects into the active site in a similar manner, the two loop types diverge significantly between residues 96 and 102 (Figure 4B).

In OXA-45, the type-II P-loop begins with an additional 3_10_ helix comprising residues 97–99 (designated helix α4a), then folds directly into helix α4, the latter helix essentially isostructural with helix α4 in the type-I P-loop. The equivalent residues (97–99) in the other structures possessing a type-II P-loop do not have the characteristics of a 3_10_ helix, with the main chain adopting a conformation closer to a type-I β-turn. The rest of the loop folds similarly to that in OXA-45 (Figure 4B). Following the common α4 helix, the type-I and type-II P-loops coincide once more as they lead into the βb_III_ anchor point.

Structural flexibility and disorder have been observed in the P-loop in several crystal structures, most notably in OXA-23, OXA-46, and OXA-146. In the OXA-23 structure solved at acidic pH in the presence of citrate (PDB code 4JF5), the P-loop is folded differently relative to the same enzyme at neutral pH (4JF6) [44]. In 4JF5, the P-loop is in an open position and has a much more expanded structure. Helix α4 is retained and is approximately 15 Å from its position in the closed form of the loop (Figure S4A). The short loop between helices α5 and α6 (*motif-3*) was also observed to be in a different, more “open” conformation [44]. A citrate molecule was tightly bound in the active site adjacent to Ser70 and Arg250, and superposition of the two OXA-23 structures suggests that it is the presence of this citrate that causes the structural rearrangement of the α5-α6 loop. This movement places the main chain of Ser118 and Ala119 directly onto the Trp105 side chain (Figure S4A), such that the tryptophan (and the entire P-loop) is forced into the open conformation. Similar movements of the α5-α6 loop are seen in OXA-46 and OXA-146, in response to tartrate- and citrate-binding, respectively. The OXA-46 structure provides us with views of the loop in two different forms within the same crystal. Monomer C has a P-loop and *motif-3* conformation isostructural with other GnDBLs with a type-I P-loop, and in this monomer, the active site is empty. Tartrate-binding in both monomers A and B induces the *motif-3* movement and triggers a conformational change in the P-loop, which is fully visible in monomer B (Figure S4B) yet completely disordered in monomer A [55].

It is notable that this side of the active site cleft is composed of flexible elements such as the α5–α6 loop and the P-loop, while the opposite side is formed by rigid structural components, including residues Ser70 and Trp211 (which form the oxyanion hole), and Thr209 and Arg250 (which anchor the C3 carboxylate). These rigid residues are responsible for the primary stabilizing hydrogen-bonding and electrostatic interactions with substrates and inhibitors. Their structural rigidity is essential for forming a fixed platform that ensures the correct positioning of the β-lactam ring for nucleophilic attack by Ser70 during formation of the Michaelis complex. In contrast, the remaining active site residues exhibit greater plasticity, allowing them to adapt their positions to accommodate variations in bound substrates and inhibitors [56].

### 3.4. The Ω-Loop

This structural feature is also common in all known GnDBLs. As noted above, *motif-5* at the center of this loop comprises a 3_10_ helix (α8), which plays a critical role in the formation of the hydrophobic cap, delineating the solvent-accessible active site from the inaccessible CLP. It was recently reported that the Ω-loop adopts at least three different conformations (types I, II, and III) in the DBLs [29]. One of these, designated the type-III Ω-loop, appears to be specific to the DBLs from *Clostridioides* and *Clostridium* sp. but not other Gram-positive DBLs, including those from *Bacillus* sp [25,53]. The other two, type-I and type-II, are seen in both Gram-positive and Gram-negative enzymes. Irrespective of the Ω-loop type, helix α8 is essentially isostructural in all the DBLs and is anchored in place via a series of internal and external hydrogen bonds, along with interactions within the hydrophobic core of Domain-2 (Figure 3D). Most importantly, the key residues Trp157 and Leu158 are consistently positioned at the edge of the CLP, maintaining their functional orientation (Figure 3F).

**Figure 5:**
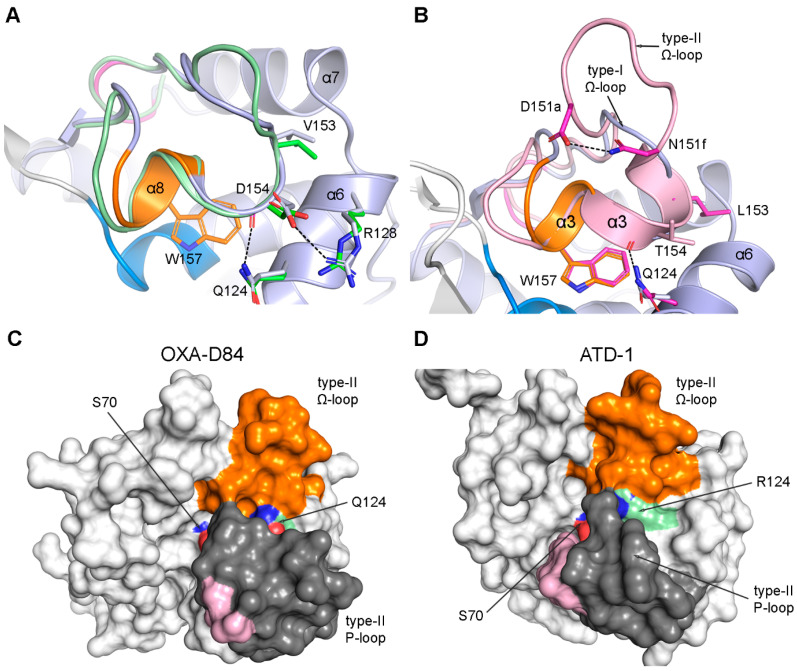
The DBL Ω-loop. (**A**) The type-I Ω-loop from OXA-23 (green ribbons and sticks) superimposed on OXA-48 (light blue ribbons and sticks). Hydrogen-bonding interactions that hold the loop against helix α6 are shown as black dashed lines. The side chain of Val153 is held in a pocket formed by the backbone of the turn connecting helices α6 and α7. (**B**) The type-II Ω-loop from OXA-1 (pink ribbons and magenta sticks) superimposed on OXA-48 (light blue ribbons and sticks). The alternate location of helix α3 in the type-II loop is indicated. The side chain of Trp157 is isostructural in both loop types. (**C**) Molecular surface of OXA-D84, which possesses both a type-II P- and Ω-loop. The two loops are connected via hydrophobic interactions and bridged via the side chain of Gln124. (**D**) Molecular surface of ATD-1 (6V6N) from *Agrobacterium tumefaciens*, also harboring type-II P- and Ω-loops, bridged by the side chain of Arg124.

The type-I Ω-loop predominates in the GnDBLs (Table S2), with 29 of the 40 enzymes having this conformation. In these enzymes, the loop (residues 144–165) begins with *motif-4* and has the same overall structure, an extended loop leading into a type-I β-turn, followed by helix α8 (*motif-5*) and β-bridge βb_IV_. The loop lies along the surface of the enzyme, and a highly conserved hydrophobic residue at position 153 (70% Val, 14% Ile, 6.5% Leu in the GnDBLs) at the tip of the loop is inserted into a hole created by the short turn joining surface helices α6 and α7 (Figure 5A). The side chains of Gln124 and Arg128 from helix α6 (both 86% conserved in the GnDBLs) interact respectively with the main chain and side chain of a highly conserved Asp154 (>90%) to anchor this part of the Ω-loop (just prior to helix α8) against the side of Domain-2 (Figure 5A). The two ends of the Ω-loop are connected via three hydrogen-bonding interactions between Asn146 from *motif-4* and residue 163 near its C-terminus. This end of the loop is in turn connected via βb_IV_ to βb_I_, and through that interaction to the N-terminus of the catalytic helix α3 (Figure 3E).

The type-II loop arises from a six-residue insertion (consensus sequence (D/E)PGKNN) within the archetypal DBL fold, occurring between residue positions 151 and 152 (denoted 151a–151f in the SAND nomenclature; Figure 2 and Figure S2), approximately four residues upstream of *motif-5* [29]. This loop extends outward from the core of the protein (Figure 5B) and includes a type-II β-turn formed by the first four residues of the insertion. A stabilizing hydrogen bond is observed between the first residue of the insertion (151a) and the last (151f), stabilizing the loop conformation. Helix α8, rather than corresponding to *motif-5* as it does in the type-I loop, begins at residue 151f. Nonetheless, Trp157 occupies an identical position in both loop types (Figure 5B). Seven enzymes possess a type-II Ω-loop: AFD-1, ATD-1, LoxA9, OXA-1, OXA-45, OXA-427, and OXA-D84. Six of these also harbor a type-II P-loop, and in some cases, the two loops are structurally connected either directly via hydrophobic interactions, such as in OXA-D84 (Figure 5C), or bridged via Gln124 or Arg124 (Figure 5D). It should be noted that the six-residue Ω-loop extension is too far (~20 Å) from the P-loop and is not itself involved in this interloop connection. Due to the current lack of substrate specificity data for most of these enzymes, the functional implications of possessing both loop types remain unclear. OXA-D84, which shares 91% sequence identity with OXA-42 and OXA-57, is classified within the OXA-42 family from *Burkholderia pseudomallei*. While these enzymes were originally designated as NSBLs [57], recent characterization of OXA-57 revealed weak but measurable carbapenemase activity [58]. This finding aligns with broader observations that many OXA enzymes exhibit at least limited activity against carbapenems [59], and raises the possibility that the co-occurrence of type-II P-loop and Ω-loop structures may contribute to this phenotype in some GnDBLs.

Four enzymes (OXA-2, OXA-46, OXA-935, and NOD-1) exhibit atypical Ω-loop extensions that do not conform to any of the three main types. As noted earlier, OXA-935 has a disordered Ω-loop due to an F156S mutation [49], and this severely disrupts enzyme activity. In OXA-2 (6XJ3), although the loop is the same length as the type-I loop, residues 151 to 155 project outward from the protein at ~90° to the archetypal type-I loop conformation (Figure S5). This divergence is likely due to the presence of a glycine at position 154 in place of the conserved aspartate, which normally tethers the loop to helix α6. As a result, several of the reported OXA-2 structures show this region to be disordered. In OXA-46 (3IF6) from *Pseudomonas aeruginosa*, the Ω-loop is also the same length as the type-I loop but adopts a distinctly different conformation. Between *motif-4* and helix α8, the loop deviates markedly from the archetypal OXA-48 type-I fold, with the β-turn preceding helix α8 absent (Figure S5A). Finally, in NOD-1 (6NHS) from the cyanobacterium *Nostoc* sp. (strain PCC 7120/SAG 25.82/UTEX 2576), the Ω-loop has a shorter two-residue insertion at position 151 (Pro151a and Glu151b). Rather than extending outward, this insertion forms an additional 3_10_ helix (designated α8′; Figure S5). The Pro151a ring stacks against the side chain of His132 at the N-terminus of helix α7, with the histidine also donating a hydrogen bond to the carbonyl oxygen of Gly150. Despite these structural differences, superposition using SSM places *motif-4*, helix α8, and β-bridge βb_IV_ in nearly identical positions across all three enzymes (Figure S5), and most importantly, Trp157 and Leu158 maintain their positions relative to the other GnDBLs and continue to contribute to the formation of the CLP and stabilization of Lys73^CO2^.

### 3.5. The B-Loop

This loop connects strands β5 and β6 and is located on the opposite side of the active site relative to the P-loop, and adjacent to the Ω-loop. Among all the structural elements in the DBLs, the B-loop displays the greatest variability in both length and conformation. Several studies have elegantly demonstrated that targeted alterations to the B-loop can significantly affect enzymatic activity. These include shortening the loop to improve substrate access to the active site [41], altering critical residues in the loop [60,61], or replacing the native loop in one enzyme with loops from other enzymes with different substrate specificities [62,63]. Collectively, these studies make it clear that the structural heterogeneity of the B-loop is a critical determinant of the broad substrate specificity observed across DBLs, underscoring its role in modulating enzyme-substrate interactions and influencing catalytic profiles.

**Figure 6:**
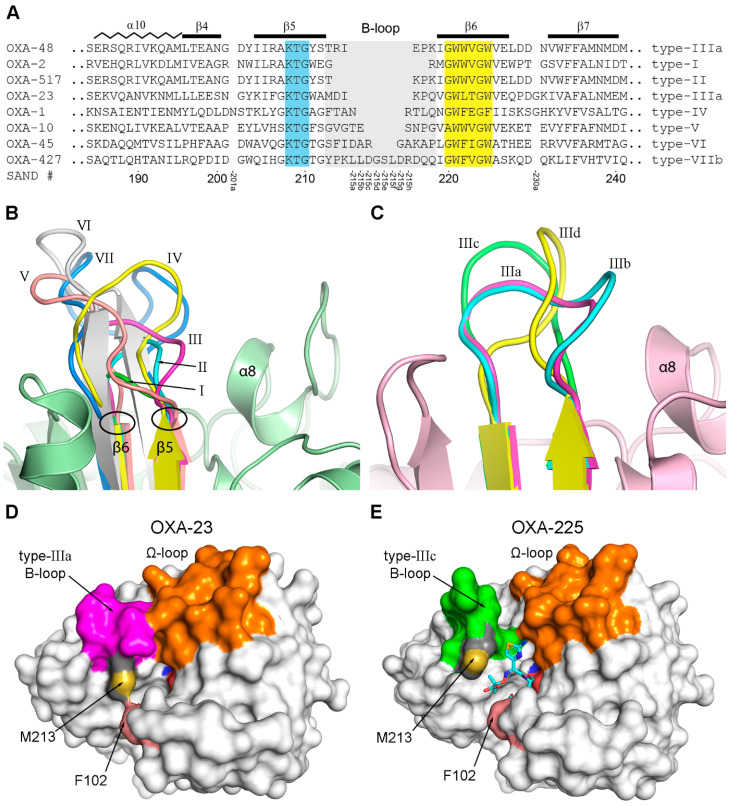
The DBL B-loop. (**A**) Segment of the structure-based multi-sequence alignment for eight GnDBLs harboring different variants of the B-loop. The two sequence motifs that bracket the loop are colored cyan (*motif-6*) and yellow (*motif-7*). The loop types are given on the right side of the alignment, and the SAND numbering scheme is given at the bottom. (**B**) The seven B-loop variants. The black circles denote the structurally invariant residues at the beginning and end of the loop. (**C**) Four variants of the type-III B-loop. (**D**) Molecular surface representation of OXA-23 showing the connectivity between the B- and Ω-loops. Met213 is indicated forming a hydrophobic bridge over the active site in conjunction with the side chain of Phe102. (**E**) Molecular surface of OXA-225, an OXA-23-like variant with a type-IIIc loop. In this structure although Met213 is present, the hydrophobic bridge is disrupted by a bound ceftazidime (cyan sticks).

Superposition of the 40 GnDBL structures (Figure S3) shows that there are at least seven major loop types (I–VII) (Figure 6A,B), along with several sub-types of the type-III loop (Table S2). The B-loop variants are named here in order of their increasing length, counting between the two structurally-conserved residues at the C- and N-termini of strands β5 and β6 (Figure 6B). The shortest loop (type-I, found in OXA-2, OXA-46, OXA-163, and OXA-405) comprises only two residues, while the longest (type-VII, found in AFD-1, ATD-1, Lox-A9, OXA-427, and OXA-D84) is composed of 14 residues. The four enzymes with the shortest type-I loop are classified as either narrow-spectrum (OXA-2 and OXA-46) or extended-spectrum β-lactamases (OXA-163 and OXA-405). Although OXA-163 and OXA-405 are members of the OXA-48 CHDL family, they exhibit negligible carbapenemase activity, primarily due to a four-residue deletion in their B-loop relative to the canonical OXA-48 type-IIIa loop [40,64,65]. Unlike the parental OXA-48, however, both variants have the ability to hydrolyze ceftazidime and cefotaxime [66,67], due to the shortened B-loop. Another OXA-48-like enzyme, OXA-517, has a type-II B-loop consisting of a two-residue deletion from the type-IIIa loop. This shortened loop, intermediate in length between the type-I and type-III loops, is sufficient to confer ESBL activity while preserving carbapenemase function [68]. The type-III B-loop is the most predominant (seen in 21 GnDBLs), comprising either six or seven residues depending on the sub-type (Figure 6C), and high-level CHDL activity amongst the GnDBLs can be directly correlated with the presence of this loop variant. This will be discussed in detail later.

Enzymes with longer loops, including OXA-1 with a type-IV loop, OXA-10 with a type-V loop, and OXA-45 with a type VI loop (Figure 6B), all lack significant CHDL activity and are classified either NSBLs or ESBLs (Table 2). These loops project outward from the molecular surface, creating a substantial gap between the B-loop and the Ω-loop (Figure S6A–C). The open conformation is capable of accommodating the bulky side chains of penicillins as well as the R2 groups of cephalosporins, thereby enabling both NSBL and ESBL activity. The longest loop (type-VII) is invariably paired with a type-II Ω-loop, and in these enzymes, there is often some connectivity between the two extended loops (Figure S6D–F). Moreover, these same enzymes also possess the alternate type-II P-loop, where connectivity to the Ω-loop has also been noted (Figure 5C,D). Of the five GnDBLs bearing a type-VII B-loop, functional data are currently available only for OXA-427. The enzyme is classified as a carbapenemase isolated from several *Enterobacteriaceae* sp. [69] and is the parental member of a new OXA family along with OXA-917. While the substrate specificities of the remaining type-VII enzymes are unknown, the coexistence of all three loop types raises the intriguing possibility that they may also possess weak carbapenemase activity.

#### The Type-III B-Loop

Fourteen GnDBLs possess the type-IIIa loop, six OXA-48-like CHDLs (OXA-48, OXA-54, OXA-181, OXA-232, OXA-245, OXA-436), plus seven *Acinetobacter* CHDLs from the OXA-23, OXA-24, OXA-51, OXA-58 and OXA-143 families, along with NOD-1 whose substrate specificity is not known (Table S2). A notable characteristic of the type-IIIa loop is the presence of a *cis*-proline at position 217 (almost universally conserved in the OXA-48-like and *Acinetobacter* enzymes) at the N-terminus of strand β6, which creates a sharper turn of the polypeptide, broadening the loop that precedes it such that residues 214 and 215 at the N-terminal end of the loop angle towards the Ω-loop (Figure 6B). It was suggested that the identity of residue 214 in the B-loop may be critical for carbapenemase activity in OXA-48 due to the presence of a salt bridge between the canonical Arg214 and Asp159 in the Ω-loop [61]. OXA-232, an OXA-48-like enzyme with serine at position 214, has diminished carbapenemase activity relative to OXA-48 primarily due to the lack of this salt bridge. In apparent contrast, the *Acinetobacter* CHDLs do not have Arg214, the equivalent residue being predominantly aspartate (in 95% of the enzymes) or asparagine, alanine, and glycine (at < 2% each). Moreover, residue 159 on the Ω-loop is a valine (>94% conserved) or isoleucine (~2%). Inspection of the superimposed *Acinetobacter* B-loops points to Val215 (>88% conserved) being involved in a hydrophobic interaction with Val159, and this interaction helps maintain contact between the two loops despite the lack of the Arg-Asp salt bridge (Figure 6D).

As noted above, the superpositions also show some type-III B-loops variants amongst the *Acinetobacter* enzymes that do not fit with the above argument. OXA-146, OXA-225, and OXA-239 (all OXA-23-like enzymes) and OXA-160 from the OXA-24 family have either type-IIIb, -IIIc, or -IIId loops. The type-IIIb loop is seen in OXA-146 and resembles the type-IIIa loop but has an extra residue due to the duplication of Ala212 (Figure 6C). This results in elevated activity against aztreonam, cefotaxime, ceftriaxone, and ceftazidime relative to the parental OXA-23 [70]. OXA-160, OXA-225, and OXA-239 have a proline-serine mutation at position 217 [66], which alters the backbone conformation of the B-loop, resulting in a more β-hairpin-like structure (classified as type-IIIc and type-IIId loops). This contrasts with the tighter turn enforced by the *cis*-proline in the type-IIIa loop. Consequently, the B-loops in these variants project outward rather than folding toward the Ω-loop, eliminating interactions with Ω-loop residues (Figure 6E). Both OXA-160 and OXA-225 retain their CHDL activity and have higher ESBL activity relative to their respective parental enzymes, OXA-24 and OXA-23. This increased ESBL activity results from the expanded cleft between the B-loop and the Ω-loop, which better accommodates the thiazole ring of ceftazidime (Figure 6E) and aztreonam. It has been suggested that the proline-to-serine mutation may have arisen as a selective adaptation in response to antibiotic pressure from these drugs [66]. The retention of CHDL activity, despite the absence of interaction between the B-loop and the Ω-loop, challenges the notion that connectivity between these loops is the primary driver of CHDL functionality. These observations from OXA-160 and OXA-225 suggest that alternative structural features may underlie CHDL activity in enzymes from different bacterial species. Although these enzymes share the conserved DBL fold (perhaps reflecting a common evolutionary origin from ancestral penicillin-binding proteins), the OXA-48 family and the *Acinetobacter* enzymes may have developed carbapenemase activity through distinct structural adaptations [71].

Although there is no strong sequence similarity in the B-loops amongst those enzymes that have the same type (except amongst those from the same OXA family), it has been noted that a number of CHDLs from *Acinetobacter* sp. have a conserved methionine residue at position 213 (Figure 2 and Figure S2), which, along with residue 102, is involved in the formation of the hydrophobic bridge over the active site (Figure 3B). This bridge was first observed in OXA-24 [42], and it has been suggested that it may be the defining factor in the CHDL activity of the *Acinetobacter* enzymes by influencing the tautomerization state of the bound carbapenem and thus promoting their hydrolysis [43,45,72]. However, in two *Acinetobacter* CHDL families (OXA-51 and OXA-213), this methionine is replaced by tryptophan at position 213. In apo-OXA-51, the tryptophan interacts with Ile101, Phe102, and Trp105 on the P-loop and creates an even more expansive hydrophobic bridge (Figure S7A) than that observed in OXA-23 or OXA-24 (Figure 6D and Figure S7B). The OXA-51 bridge is so large, in fact, that it would significantly occlude the active site. Although no crystal structure is currently available for any member of the OXA-213 family, AlphaFold2 modeling of the parental OXA-213 enzyme [29] revealed a similarly broad, hydrophobic bridge that likely perturbs substrate access (Figure S7C). Since these enzymes are efficient β-lactamases and also possess CHDL activity, it was suggested that a change to an alternate Trp213 rotamer would allow for carbapenem access into the active site [54], and it was subsequently shown that this was indeed the case [56]. Furthermore, it was demonstrated that mutation of Trp213 to Met213 to more closely resemble other *Acinetobacter* CHDLs increased the MICs of carbapenems 8-fold, and the catalytic efficiency of the Trp213Met mutant against the same carbapenems increased 10-fold, bringing the activity in line with OXA-23 and OXA-24 [54].

Since the OXA-48-like CHDLs lack this structural feature (with threonine invariably present at position 213), the carbapenemase activity observed in *Acinetobacter* OXA enzymes could derive from a combination of the presence of a type-IIIa B-loop and some degree of connectivity with the Ω-loop, along with the addition of the hydrophobic bridge. Notwithstanding this, the similarity in B-loop conformations between OXA-48 and *Acinetobacter* CHDLs, combined with evidence that even minor alterations in the sequence or structure of this loop can markedly influence enzyme specificity, still strongly supports the identification of the B-loop as a major determinant of substrate specificity.

### 3.6. Dynamics and Stability Within the DBL Fold

While the crystal structures of DBLs have been instrumental in elucidating the general architecture and active site features of these enzymes, they offer only static snapshots of what are inherently dynamic enzymes. There is often a temptation to interpret these structures as complete representations of function, somewhat overlooking the crucial role of conformational flexibility in substrate recognition, catalysis and inhibitor binding. In reality, the DBLs likely operate through an ensemble of interconverting conformational states, involving loop motions and side chain rearrangements that facilitate the accommodation of diverse β-lactam substrates. This dynamic behavior may be particularly relevant in the DBLs, where subtle motions of the active site loops, such as the P-loop, the α5-α6 loop, the Ω-loop, and the B-loop, could modulate activity and specificity. We have seen evidence of flexibility in the first three structural elements, and the presence of a multitude of different structures of the B-loop hints at its dynamic nature. This underscores the need to complement the crystallographic data on the DBLs with approaches that capture protein motion.

One of the simplest indicators of protein dynamics can be derived from static crystal structures themselves. Atomic displacement parameters (ADPs), also known as B-factors, quantify the extent to which each atom deviates from its average position, reflecting thermal motion and other sources of positional uncertainty within the electron density. Regions of the protein with elevated ADPs are generally more flexible or dynamic, while regions with lower ADPs tend to be more rigid. In addition to crystallographic data, molecular dynamics (MD) simulations offer an alternative and more comprehensive approach to visualizing and analyzing protein flexibility by modeling atomic motions over time.

**Figure 7:**
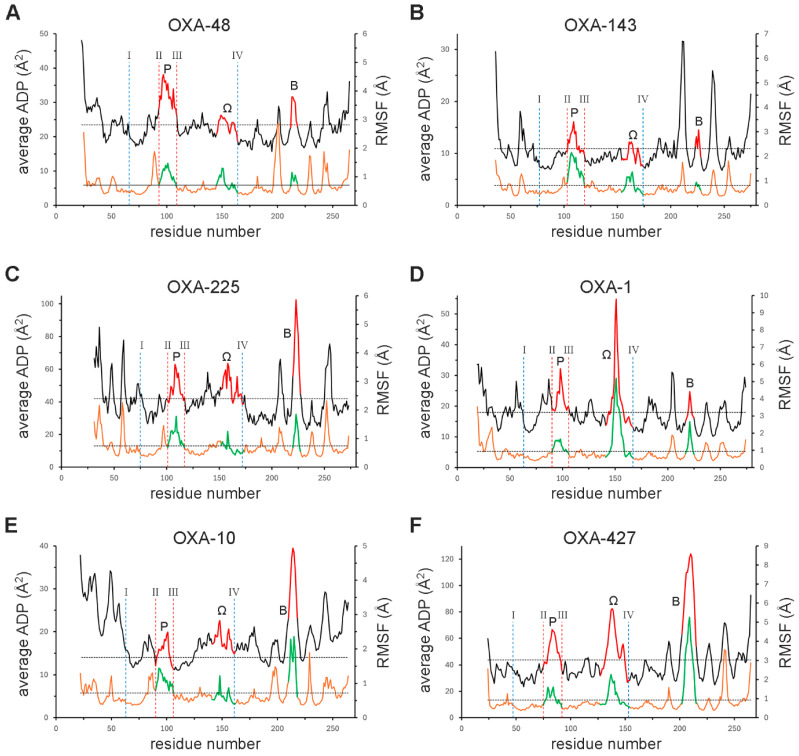
Average ADP and RMSF as a function of residue number for selected GnDBLs. (**A**) OXA-48. (**B**) OXA-143. (**C**) OXA-225. (**D**) OXA-1. (**E**) OXA-10. (**F**) OXA-427. The ADP plot is the top black trace and the RMSF plot if the lower orange trace. The P-loop, Ω-loop and B-loop are highlighted in red and green on the two traces. The positions of the four β-bridges are given as vertical dashed blue (βb_I_ and βb_IV_) and red (βb_II_ and βb_III_) lines. Horizontal dashed lines represent the average values for the ADP and RMSF plots.

To visualize structural flexibility in the GnDBLs, ADPs were extracted from crystal structures and plotted as a function of residue number for a representative selection of enzymes (black traces; Figure 7A–F). To complement this static view, a series of 250 ns molecular dynamics (MD) simulations was performed on the same set of GnDBLs to provide a more complete picture of their conformational flexibility. From the resultant MD trajectories, the root-mean-square fluctuation (RMSF), the average positional deviation of each residue from its mean location over time, was calculated for each residue (orange traces; Figure 7A–F). This allowed for the identification of regions within the enzymes that are more flexible (high RMSF) or more rigid (low RMSF). While these 250 ns MD simulations offer valuable insights into protein flexibility, they may not fully capture any slower, large-scale conformational changes. Acknowledging this limitation, future studies employing longer trajectories or enhanced sampling techniques such as metadynamics or accelerated MD may be necessary to more comprehensively explore the conformational landscape of these enzymes.

For OXA-48, the overall average main chain ADP and RMSF are 23.4 Å^2^ and 0.7 Å, respectively (Figure 7A). The regions of high and low flexibility are consistent across both measurements, supporting the utility of ADPs as a reasonable proxy for assessing residue-level flexibility in this enzyme. As expected, well-ordered secondary structure elements exhibit lower ADPs and fluctuations, whereas loops, turns, and the N- and C-termini tend to be more flexible. The P-loop, Ω-loop, and B-loop are highlighted in red and green on the ADP and RMSF traces, respectively (Figure 7A). Each of the three loops in OXA-48 shows motion levels approximately 1.5-fold greater than the overall average. Notably, the Ω-loop exhibits dimorphic behavior; its N-terminal portion (residues 147–155) is significantly more flexible than the C-terminal segment (156–164). This difference likely arises from the presence of helix α8 and the network of stabilizing interactions involving the βb_IV_ and βb_I_ β-bridges, as well as the B-loop, which collectively contribute to the rigidity of the latter half of the loop.

A similar pattern is also observed in the three loops in OXA-143 (Figure 7B) and in OXA-225 (Figure 7C). In OXA-143, the P-loop as well as the Ω-loop show differing levels of flexibility from one end to the other. In the P-loop, the α4 helix has much lower fluctuations than the earlier section of the loop, once again a consequence of the internal hydrogen-bonding in the helix. As with OXA-48, the type-IIIa B-loop in OXA-143 exhibits very limited motion, likely resulting from the interlocking of the B-loop and the Ω-loop, which stabilizes this region in both enzymes. The type-IIIc loop in OXA-225, however, shows an increased level of flexibility (Figure 7C), over 2-fold higher than the average, reflecting the lack of stabilizing contacts with the Ω-loop (Figure 6E).

A significant increase in Ω-loop flexibility is observed in OXA-1 (Figure 7D), a non-CHDL enzyme with a type-II Ω-loop. The fluctuation data show that the six-residue insertion within this loop exhibits the highest dynamic behavior among all the enzymes analyzed by MD. The MD analysis also suggests some increased motion in the type-IV B-loop comparable to that seen in OXA-225, in contrast to the shorter, more constrained type-IIIa loop in OXA-48 and OXA-143. Inspection of the OXA-1 structure shows an absence of stabilizing contacts between the B-loop and the Ω-loop (Figure S6A), which may contribute to the observed flexibility. OXA-10 displays Ω-loop fluctuations comparable to those in OXA-48 and OXA-143 (Figure 7E), but its type-V B-loop is nearly three times more flexible than average, likely due to the lack of stabilizing interactions between the elongated loop and the surrounding protein surface (Figure S6B). Some of the highest levels of motion across all three loops are seen in OXA-427 (Figure 7F). As in OXA-1, the type-II Ω-loop in OXA-427 exhibits dimorphic behavior, with the six-residue insertion showing nearly twice the mobility of the α8 helix.

### 3.7. The β-Bridges

The ADP and RMSF plots also reveal that the four β-bridges are among some of the least dynamic elements of the structures (Figure 7), despite occurring either within short turns (βb_I_ and βb_IV_) or in association with the flexible P-loop (βb_II_ and βb_III_). Notably, βb_I_ and βb_IV_ exhibit fluctuations comparable to those of adjacent helices, highlighting their structural rigidity. This is particularly notable given recent suggestions that these β-bridges are transient and potentially not conserved across all DBLs [30]. Examination of the 40 GnDBL structures shows that these β-bridges are always present, and the hydrogen-bonding between them is conserved. In the majority of cases where conformational differences or disorder are observed in the P-loop or Ω-loop, two hydrogen-bonding interactions between the paired β-bridges (βb_II_ and βb_III_; residues 93 and 109, and βb_I_ and βb_IV_; residues 66 and 164) over the course of the 250 ns MD trajectories remain intact (Figure S8). The exception is the low pH citrate-bound form of OXA-23, where the loop conformation is different at Met109 such that the Phe93N–Met109O bond is present but the Phe93O–Met109N bond cannot form.

In the βb_II_–βb_III_ pairing, residue 109 is locked against the N-terminus of helix α5, and the two hydrogen bonds with residue 93 thus provide a strong anchor point at the base of the P-loop. As noted earlier, this loop is a key functional element on the adaptive side of the DBL active site, and its ability to adjust its position relative to the fixed side of the cleft (strand β5 and the N-terminus of helices α3 and α11) is essential for enzymatic function [56,73]. Flexibility provided by the βb_II_–βb_III_ hinge point facilitates this movement, which is reflected in the fluctuation analyses, with residues 93 and 109 showing fluctuations close to the molecular average, indicating moderate mobility, while the loop between shows increased movement (Figure 7). In contrast, the residues forming the βb_I_ and βb_IV_ pairing exhibit significantly lower fluctuations than the molecular average, consistent with their role as a rigid anchor and their location at the edge of the Domain-2 hydrophobic core (Figure 3D). This β-bridge immobilizes *motif-5*, ensuring proper positioning of Trp157 and Leu158, critical for formation of the CLP and stabilization of Lys73^CO2^, irrespective of the conformational dynamics of the Ω-loop that precedes it. These findings reinforce the idea that, at least in the GnDBLs analyzed here, the β-bridges contribute more to structural stability and conservation than previously assumed. Given their strategic positions within the DBL architecture and in light of the dynamic properties of surrounding surface loops, these short structural elements play a vital role in substrate-binding and catalytic function.

## 4. Concluding Remarks

The DBL fold comprises a structurally conserved two-domain architecture, with each domain centered around a hydrophobic core, and includes seven essential sequence motifs and three adaptable surface loops. Together, these elements govern substrate recognition, catalytic activity, and active site mechanics. The P-loop functions as a pliable, conformationally flexible boundary along one edge of the active site cavity, enabling accommodation of substrates with diverse structural features. In contrast, the Ω-loop lends structural stability to the active site, anchoring key elements such as Ser70 and Lys73^CO2^ to maintain catalytic efficiency. The B-loop has emerged as a principal determinant of substrate specificity, particularly for carbapenems, due to its variable length, conformational plasticity, and interactions with adjacent structural elements, including the Ω-loop and the hydrophobic bridge found in certain CHDLs. The coordinated flexibility of these three loops provides the molecular foundation for the DBL family’s ability to evolve substrate specificity while maintaining catalytic competence. This mechanism represents a fundamental driver of persistent β-lactam resistance emergence among clinically significant Gram-negative bacteria, underscoring the ongoing challenge DBLs pose to antimicrobial therapy.

Elucidating the concerted structural and dynamic roles of the P-loop, Ω-loop, and B-loop provides a detailed framework for understanding how the DBLs adapt to and hydrolyze a broad range of β-lactam antibiotics, including last-resort carbapenems. Importantly, the identification of the B-loop as a key determinant of substrate specificity presents a promising new target for structure-guided inhibitor design. Strategies that interfere with the coordinated motion or interactions among these loops, or that stabilize inactive conformations by targeting conserved structural features, may provide effective countermeasures against DBL-mediated resistance. In particular, the interface between the B-loop and Ω-loop, essential for carbapenemase activity in *Acinetobacter* GnDBLs and OXA-48-like enzymes, represents a key vulnerability. Compounds designed to destabilize this loop interface could function as effective carbapenemase inhibitors. Moreover, inducing increased flexibility or disorder in the Ω-loop itself would drastically impair deacylation by dismantling the CLP and exposing Lys73^CO2^ to the solvent. This would effectively convert all β-lactam substrates into irreversible covalent inhibitors and thereby broadly suppress DBL activity. Together, these mechanistic insights offer a foundation for rational design of next-generation β-lactamase inhibitors and allosteric modulators aimed at restoring β-lactam efficacy against multidrug-resistant Gram-negative pathogens.

## Figures and Tables

**Table 1:** The class D fingerprint sequence motifs.

Motif	Consensus Sequence *^a^*	Sequence Identity at Variable Positions *^b^*	Residue Numbering *^c^*
1	PASTFK	1:P 99.3% (T, S, A and Q < 0.7%) 2:A 95.3%, E 1.2%, D 1%, C 0.8%, Q 0.8% (M, V, G, T and Y < 1%) 4:T 99.5%, (S and I < 0.5%) 5:F 93%, Y 6.9%	68–73
2	FxxW	1:F 64.1%, L 9.5%, I 7.8%, M 6.6%, G 5.4%, Y 2.7%, (N, V, R, S, C, P and T < 3.9%) 4:W 96.2%, H 3.3% (T and C < 0.5%)	102–105
3	SAV	1:S 99.9% (P and Y < 0.1%) 2:A 61.8%, V 16%, N 9.5%, Q 5.1%, T 4%, C 3.3%, I 0.3% 3:V 56.2%, I 40.1%, L 2.1%, F 0.9% (Y, M and A < 0.7%)	118–120
4	YGN	1:Y 77.7%, F 22.2% 2:G 99.6% (S, D, K and R < 0.4%) 3:N 99% (E, K, A, S, D, G, V and Y < 1%)	144–146
5	FWL	1:F 85.8%, A 8.1%, V 4.7%, S 1.2% 2:W 99% (G, C, L, R and D < 1%) 3:L 87.5%, I 8.9%, V 2.8% (H, Q, E and K < 0.7%)	156–158
6	K(S/T)**G**	1:K 99.7% (R, E and Q < 0.3%) 2:S 52.3%, T 47.6%	208–210
7	GWx(T/V)**G**W	1:G 87.5%, A 12.2% (S, C and T < 0.3%) 2:W 99.6% (Q, G and R < 0.4%) 4:T 55.2%, V 42.3%, E 1%, I 0.9%, A 0.5% (F and S < 0.1%) 6:W 87.1%, F 10.3%, Y 1.9% (M, H, I, L, S and V < 0.7%	220–225

*^a^* Amino acids in bold are 100% conserved in all GnDBL sequences. *^b^* The numbers indicate the position of the particular amino acid in the motif. *^c^* Standard DBL numbering [29,30].

**Table 2:** B-loop variants and enzymatic activity profiles.

B-Loop Type	Variants	Residues	Enzyme(s)	Activity
I	-	2	OXA-2 *^a^*	NSBL, ESBL
II	-	4	OXA-517	CHDL, ESBL
III	a	6	OXA-23 *^b^*	CHDL
III	b	7 *^c^*	OXA-146	CHDL
III	c	6	OXA-160 *^d^*	CHDL
III	d	6	OXA-239	CHDL
III	e	7	OXA-85	NSBL
IV	-	8	OXA-1	NSBL
V	-	9	OXA-10 *^e^*	ESBL
VI	-	11	OXA-45	ESBL
VII	a	14	OXA-D84 *^f^*	Unknown
VII	b	14	OXA-427	CHDL

*^a^* Also OXA-46, OXA-163 and OXA-405. *^b^* Also OXA-24, OXA-48, OXA-51, OXA-54, OXA-58, OXA-66, OXA-143, OXA-181, OXA-231, OXA-232, OXA245, OXA-436 and NOD-1. *^c^* Due to a duplication of Ala212. *^d^* Also OXA-225, CPD-1 and STD-1. *^e^* Also OXA-13, OXA-14, OXA-17, OXA-145, OXA-665 and OXA-935. *^f^* Also AFD-1, ATD-1 and Lox-A9.

## Data Availability

All the data generated during this work are contained in this article and Supplementary Materials.

## Supplementary Materials

The following supporting information can be downloaded at: https://www.mdpi.com/article/10.3390/pathogens14080761/s1, Figure S1. Histogram of the numbers of annotated β-lactamase enzymes in the four molecular classes. Figure S2. Structure-based sequence alignment of Gram-negative DBLs. Figure S3. Structural superposition of the Gram-negative DBLs. Figure S4. DBL P-loop flexibility. Figure S5. Atypical Ω-loops in the GnDBLs. Figure S6. Molecular surface representations of several GnDBLs. Figure S7. Molecular surface representations. Figure S8. Hydrogen-bonding in the β-bridges. Table S1. GnDBL enzyme families. Table S2. GnDBL structures.

## Abbreviations

DBL, class D β-lactamase; NSBL, narrow spectrum β-lactamase; ESBL, extended-spectrum β-lactamase; CHDL, carbapenem-hydrolyzing class D β-lactamase; GnDBL, Gram-negative DBL; BLDB, β-lactamase Database, PDB, Protein Data Bank; SSM, secondary structure matching; MD, molecular dynamics; CLP, catalytic lysine pocket; DWP, deacylating water pocket; ADPs, atomic displacement parameters; RMSF, root-mean-square fluctuation.
