# Supplementary material for: Diversity in the Common Fold: Structural Insights into Class D β-Lactamases from Gram-Negative Pathogens
Source: Pathogens. 2025 Aug 1;14(8):761. doi: 10.3390/pathogens14080761 (PMC12389319; doi:10.3390/pathogens14080761)
Supplement: Supplementary file 1 [file pathogens-14-00761-s001.zip › pathogens-3761291-supplementary.pdf]

Supplementary Material for

Diversity in the Common Fold: Structural Insights into Class D β-Lactamases from Gram-Negative Pathogens

Clyde A. Smith[1,2*] and Anastasiya Stasyuk[1,3]

[1] Stanford Synchrotron Radiation Lightsource, SLAC National Accelerator Laboratory, Menlo Park, CA 94025, USA

[2] Department of Chemistry, Stanford University, Stanford, CA 94303, USA

[3] University of California Davis Medical Center, Sacramento, CA 95817, USA

[*]Address correspondence to this author:

Dr Clyde A. Smith, ph: +1-650-926-8544, email: csmith@slac.stanford.edu

Supplementary Figures

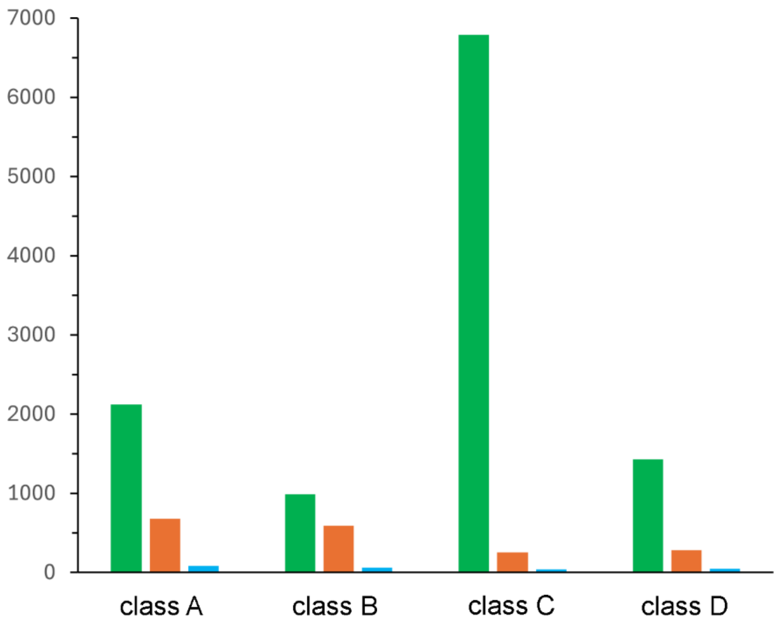

Figure S1. Histogram of the numbers of annotated β-lactamase enzymes in the four molecular classes. The green bars denote the total number of known sequences, the orange bars are the total number of structures (including mutants and substrate/inhibitor complexes) and the blue bars represent the number of unique structures. The data was extracted from the β-Lactamase Database (BLDB; www.bldb.eu) [1] accessed 20 June 2025.

```
                β1     α1           β2            β3         α2    βbI       α3
OXA-1          STDISTVASPLFE--GTEGCFLLYDAS  TNAEIAQF  NKAKCATQMAPDSTFKIALSLMAFDAEII-DQKT
OXA-2          TLER SDWRKFFSEFQAKGTIVVADERQADRAM LVF  DPVRSKKRYSPASTFKIPHTLFALDAGAVRDEFQ
OXA-10        SITEN TSWNKEFSAEAVNGVFVLCKSS  SKSC ATN  DLARASKEYLPASTFKIPNAIIGLETGVIKNEHQ
OXA-13        SITEN TSWNKEFSAEAVNGVFVLCKSS  SKSC ATN  NLARASKEYLPASTFKIPSAIIGLETGVIKNEHQ
OXA-14        SITEN TSWNKEFSAEAVNGVFVLCKSS  SKSC ATN  DLARASKEYLPASTFKIPNAIIGLETGVIKNEHQ
OXA-17        SITEN TSWNKEFSAEAVNGVFVLCKSS  SKSC ATN  DLARASKEYLPASTFKIPSAIIGLETGVIKNEHQ
OXA-23        IVQGHNQVIHQYFDEKNTSGVLVIQTD-  -KKI NLYGNALSRANTEYVPASTFKMLNALIGLENQKT-DINE
OXA-24      HISSQQHEKAIKSYFDEAQTQGVIIIKEG-  -KNL STYGNALARANKEYVPASTFKMLNALIGLENHKA-TTNE
OXA-45                       QMLECTLVADAA  SGQELYRK  GA--CDKAFAPMSTFKVPLAVMGYDAGILVDAHN
OXA-46         VVIR SDWKKFFSDLQAEGAIVIADERQAKHTL SVF  DQERAAKRYSPASTFKIPHTLFALDADAVRDEFQ
OXA-48         WQEN KSWNAHFTEHKSQGVVVLWNEN  KQQG FTN  NLKRANQAFLPASTFKIPNSLIALDLGVVKDEHQ
OXA-51         SDEKAEKIKNLFNEVHTTGVLVIQQG-  -QTQ QSYGNDLARASTEYVPASTFKMLNALIGLEHHKA-TTTE
OXA-54         WQEK PSWNTHFSEHKAQGVIVLWNEN  KQQG FTN  NLKRANQAFLPASTFKIPNSLIALDLGVVKDEHQ
OXA-58          SIIDQNVQALFNEISADAVFVTYDG-  -QNI KKYGTHLDRAKTAYIPASTFKIANALIGLENHKA-TSTE
OXA-66         SDVKAEKIKNLFNEAHTTGVLVIQQG-  -QTQ QSYGNDLARASTEYVPASTFKMLNALIGLEHHKA-TTTE
OXA-85     IISFGNEN QFMKEIFERKGLNGTFVVYDLK  NDKI DYY  NLDRANERFYPASSFKIFNTLIGLENGIVKNVDE
OXA-143        NQQHEKAIKSYFDEAQTQGVIIIKKG-  -KNI STYGNNLTRAHTEYVPASTFKMLNALIGLENHKA-TTTE
OXA-145       SITEN TSWNKEFSAEAVNGVFVLCKSS  SKSC ATN  NLARASKEYLPASTFKIPNAIIGLETGVIKNEHQ
OXA-146       IVQGHNQVIHQYFDEKNTSGVLVIQTD-  -KKI NLYGNALSRANTEYVPASTFKMLNALIGLENQKT-DINE
OXA-160     HISSQQHEKAIKSYFDEAQTQGVIIIKEG-  -KNL STYGNALARANKEYVPASTFKMLNALIGLENHKA-TTNE
OXA-163        WQEN KSWNAHFTEHKSQGVVVLWNEN  KQQG FTN  NLKRANQAFLPASTFKIPNSLIALDLGVVKDEHQ
OXA-181        WQEN KSWNAHFTEHKSQGVVVLWNEN  KQQG FTN  NLKRANQAFLPASTFKIPNSLIALDLGVVKDEHQ
OXA-225       IVQGHNQVIHQYFDEKNTSGVLVIQTD-  -KKI NLYGNALSRANTEYVPASTFKMLNALIGLENQKT-DINE
OXA-231        NQQHEKAIKSYFDEAQTQGVIIIKKG-  -KNI STYGNNLTRAHTEYVPASTFKMLNALIGLENHKA-TTTE
OXA-232        WQEN KSWNAHFTEHKSQGVVVLWNEN  KQQG FTN  NLKRANQAFLPASTFKIPNSLIALDLGVVKDEHQ
OXA-239       IVQGHNQVIHQYFDEKNTSGVLVIQTD-  -KKI NLYGNALSRANTEYVPASTFKMLNALIGLENQKT-DINE
OXA-245        WQEN KSWNAHFTEHKSQGVVVLWNEN  KQQG FTN  NLKRANQAFLPASTFKIPNSLIALDLGVVKDEHQ
OXA-405        WQEN KSWNAHFTEHKSQGVVVLWNEN  KQQG FTN  NLKRANQAFLPASTFKIPNSLIALDLGVVKDEHQ
OXA-427                       ATGCMLFADG-  SGKPFSAQ  GD--CASQLPPASTFKIPLALMGYDSGFLVDEQL
OXA-436        WQEN KSWNAHFSEHKTQGVVVLWNEN  TQQG FTN  DLKRANQAFLPASTFKIPNSLIALDLGVVKDEHQ
OXA-517        WQEN KSWNAHFTEHKSQGVVVLWNEN  KQQG FTN  NLKRANQAFLPASTFKIPNSLIALDLGVVKDEHQ
OXA-655       SITEN MSWNKEFSAEAVNGVFVLCKSS  SKSC ATN  DLARASKEYLPASTFKIPNAIIGLETGVIKNEHQ
OXA-935       SITEN TSWNKEFSAEAVNGVFVLCKSS  SKSC ATN  DLARASKEYLPASTFKIPNAIIGLETGVIKNEHQ
OXA-D84                   SNAKTICTAIADAG  TGKLLLQD  GD--CSRRASPASTFKIAISLMGYDAGFLRNEHD
AFD-1                    QQPQAFECTLVTSIE  TGAIINQQ  GA--CDQRVAPASTFKVPLALIGFDAGILQDGKT
ATD-1                    QQSQAFECTLVTSIE  TGAVINQQ  GA--CDQRVAPASTFKVPLALIGYDAGILLDDKT
CPD-1          VENE KSWEKYFAEYKVEGCFMLFNNS  QGTF KVY  NLERSQQRFLPASTFKIFNSLVGLETGVIKDTSF
Lox-A9                        QDTCFLAKEN-  -QTVLKRE  GN-DCDQRYSPASTFKIALSLMGFDSGILKDELH
NOD-1             N VNLGRSFNQLGIKGSILIYDRN  NKKF YEH  NAARNSQSFLPASTFKIFNSLVALETGVISNDVA
STD-1             N VNLQKIFDENKITGSVTIYDYK  NKIW IYS  NEEDSKIRRLPASTFKIPNSLIFLEEEVVKDENE
                -28a                   -50b -50a -54a -57b -57a
consensus                                             PASTFK
SAND #             30        40        50        60        70        80        90
```

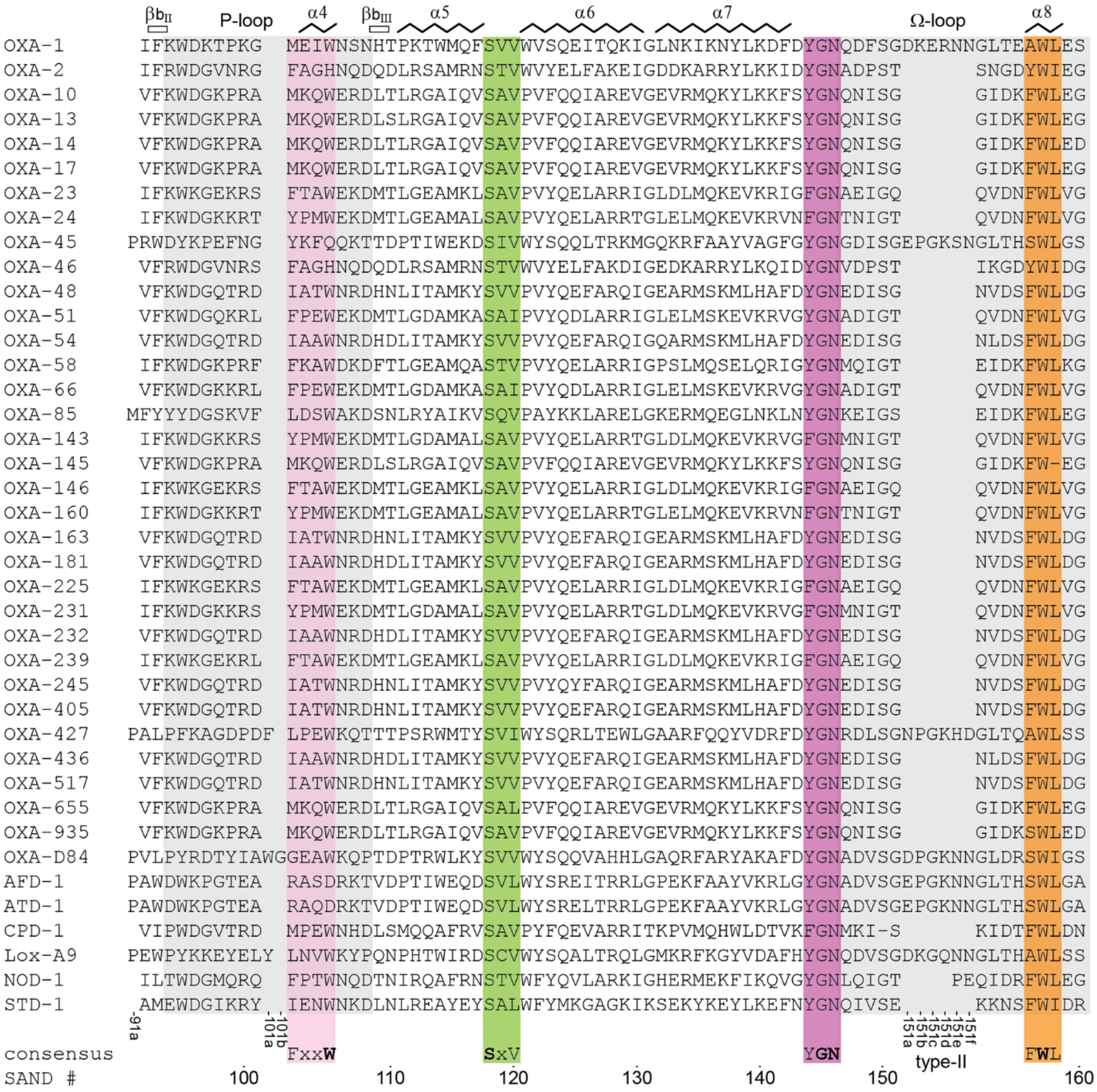
βb$_{II}$
P-loop
α4
βb$_{III}$
α5
α6
α7
Ω-loop
α8
OXA-1 IFKWDKTPKG MEIWNSNHTPKTWMQFSVVWVSQEITQKIGLNKIKNYLKDFDYGNQDFSGDKERNNGLTEAWLES
OXA-2 IFRWDGVNRG FAGHNQDQDLRSAMRNSTVWVYELFAKEIGDDKARRYLKKIDYGNADPST SNGDYWIEG
OXA-10 VFKWDGKPRA MKQWERDLTLRGAIQVSAVPVFQQIAREVGEVRMQKYLKKFSYGNQNISG GIDKFWLEG
OXA-13 VFKWDGKPRA MKQWERDLSLRGAIQVSAVPVFQQIAREVGEVRMQKYLKKFSYGNQNISG GIDKFWLEG
OXA-14 VFKWDGKPRA MKQWERDLTLRGAIQVSAVPVFQQIAREVGEVRMQKYLKKFSYGNQNISG GIDKFWLED
OXA-17 VFKWDGKPRA MKQWERDLTLRGAIQVSAVPVFQQIAREVGEVRMQKYLKKFSYGNQNISG GIDKFWLEG
OXA-23 IFKWKGEKRS FTAWEKDMTLGEAMKLSAVPVYQELARRIGLDLMQKEVKRIGFGNAEIGQ QVDNFWLVG
OXA-24 IFKWDGKKRT YPMWEKDMTLGEAMALSAVPVYQELARRTGLELMQKEVKRVNFGNTNIGT QVDNFWLVG
OXA-45 PRWDYKPEFNG YKFQQKTTDPTIWEKDSIVWYSQQLTRKMGQKRFAAYVAGFGYGNGDISGEPGKSNGLTHSWLGS
OXA-46 VFRWDGVNRS FAGHNQDQDLRSAMRNSTVWVYELFAKDIGEDKARRYLKQIDYGNVDPST IKGDYWIDG
OXA-48 VFKWDGQTRD IATWNRDHNLITAMKYSVVPVYQEFARQIGEARMSKMLHAFDYGNEDISG NVDSFWLDG
OXA-51 VFKWDGQKRL FPEWEKDMTLGDAMKASAIPVYQDLARRIGLELMSKEVKRVGYGNADIGT QVDNFWLVG
OXA-54 VFKWDGQTRD IAAWNRDHDLITAMKYSVVPVYQEFARQIGQARMSKMLHAFDYGNEDISG NLDSFWLDG
OXA-58 IFKWDGKPRF FKAWDKDFTLGEAMQASTVPVYQELARRIGPSLMQSELQRIGYGNMQIGT EIDKFWLKG
OXA-66 VFKWDGKKRL FPEWEKDMTLGDAMKASAIPVYQDLARRIGLELMSKEVKRVGYGNADIGT QVDNFWLVG
OXA-85 MFYYYDGSKVF LDSWAKDSNLRYAIKVSQVPAYKKLARELGKERMQEGLNKLNYGNKEIGS EIDKFWLEG
OXA-143 IFKWDGKKRS YPMWEKDMTLGDAMALSAVPVYQELARRTGLDLMQKEVKRVGFGNMNIGT QVDNFWLVG
OXA-145 VFKWDGKPRA MKQWERDLSLRGAIQVSAVPVFQQIAREVGEVRMQKYLKKFSYGNQNISG GIDKFW-EG
OXA-146 IFKWKGEKRS FTAWEKDMTLGEAMKLSAVPVYQELARRIGLDLMQKEVKRIGFGNAEIGQ QVDNFWLVG
OXA-160 IFKWDGKKRT YPMWEKDMTLGEAMALSAVPVYQELARRTGLELMQKEVKRVNFGNTNIGT QVDNFWLVG
OXA-163 VFKWDGQTRD IATWNRDHNLITAMKYSVVPVYQEFARQIGEARMSKMLHAFDYGNEDISG NVDSFWLDG
OXA-181 VFKWDGQTRD IAAWNRDHDLITAMKYSVVPVYQEFARQIGEARMSKMLHAFDYGNEDISG NVDSFWLDG
OXA-225 IFKWKGEKRS FTAWEKDMTLGEAMKLSAVPVYQELARRIGLDLMQKEVKRIGFGNAEIGQ QVDNFWLVG
OXA-231 IFKWDGKKRS YPMWEKDMTLGDAMALSAVPVYQELARRTGLDLMQKEVKRVGFGNMNIGT QVDNFWLVG
OXA-232 VFKWDGQTRD IAAWNRDHDLITAMKYSVVPVYQEFARQIGEARMSKMLHAFDYGNEDISG NVDSFWLDG
OXA-239 IFKWKGEKRL FTAWEKDMTLGEAMKLSAVPVYQELARRIGLDLMQKEVKRIGFGNAEIGQ QVDNFWLVG
OXA-245 VFKWDGQTRD IATWNRDHNLITAMKYSVVPVYQYFARQIGEARMSKMLHAFDYGNEDISG NVDSFWLDG
OXA-405 VFKWDGQTRD IATWNRDHNLITAMKYSVVPVYQEFARQIGEARMSKMLHAFDYGNEDISG NVDSFWLDG
OXA-427 PALPFKAGDPDF LPEWKQTTTPSRWMTYSVIWYSQRLTEWLGAARFQQYVDRFDYGNRDLSGNPGKHDGLTQAWLSS
OXA-436 VFKWDGQTRD IAAWNRDHDLITAMKYSVVPVYQEFARQIGEARMSKMLHAFDYGNEDISG NLDSFWLDG
OXA-517 VFKWDGQTRD IATWNRDHNLITAMKYSVVPVYQEFARQIGEARMSKMLHAFDYGNEDISG NVDSFWLDG
OXA-655 VFKWDGKPRA MKQWERDLTLRGAIQVSALPVFQQIAREVGEVRMQKYLKKFSYGNQNISG GIDKFWLEG
OXA-935 VFKWDGKPRA MKQWERDLTLRGAIQVSAVPVFQQIAREVGEVRMQKYLKKFSYGNQNISG GIDKSWLED
OXA-D84 PVLPYRDTYIAWGGEAWKQPTDPTRWLKYSVVWYSQQVAHHLGAQRFARYAKAFDYGNADVSGDPGKNNGLDRSWIGS
AFD-1 PAWDWKPGTEA RASDRKTVDPTIWEQDSVLWYSREITRRLGPEKFAAYVKRLGYGNADVSGEPGKNNGLTHSWLGA
ATD-1 PAWDWKPGTEA RAQDRKTVDPTIWEQDSVLWYSRELTRRLGPEKFAAYVKRLGYGNADVSGEPGKNNGLTHSWLGA
CPD-1 VIPWDGVTRD MPEWNHDLSMQQAFRVSAVPYFQEVARRITKPVMQHWLDTVKFGNMKI-S KIDTFWLDN
Lox-A9 PEWPYKKEYELY LNVWKYPQNPHTWIRDSCVWYSQALTRQLGMKRFKGYVDAFHYGNQDVSGDKGQNNGLTHAWLSS
NOD-1 ILTWDGMQRQ FPTWNQDTNIRQAFRNSTVWFYQVLARKIGHERMEKFIKQVGYGNLQIGT PEQIDRFWLEG
STD-1 AMEWDGIKRY IENWNKDLNLREAYEYSALWFYMKGAGKIKSEKYKEYLKEFNYGNQIVSE KKNSFWIDR
-91a
-101a
-101b
-151a -151b -151c -151d -151e -151f
consensus FxxW SxV YGN FWL
type-II
SAND # 100 110 120 130 140 150 160

```
             βbIV    α9                 α10        β4      β5        B-loop          β6
OXA-1      SLKISPEEQIQFLRKIINHNLPVKNSAIENTIENMYLQDLDNSTKLYGKTGAG FTAN       RTLQNGWFEGFIIS
OXA-2      SLAISAQEQIAFLRKLYRNELPFRVEHQRLVKDLMIVEAGR NWILRAKTGWE G--        --RMGWWVGWVEW
OXA-10     QLRISAVNQVEFLESLYLNKLSASKENQLIVKEALVTEAAP EYLVHSKTGFS GVGTE      SNPGVAWWVGWVEK
OXA-13     QLRISAVNQVEFLESLFLNKLSASKENQLIVKEALVTEAAP EYLVHSKTGFS GVGTE      SNPGVAWWVGWVEK
OXA-14     QLRISAVNQVEFLESLYLNKLSASKENQLIVKEALVTEAAP EYLVHSKTGFS GVGTE      SNPGVAWWVGWVEK
OXA-17     QLRISAVNQVEFLESLYLNKLSASKENQLIVKEALVTEAAP EYLVHSKTGFS GVGTE      SNPGVAWWVGWVEK
OXA-23     PLKVTPIQEVEFVSQLAHTQLPFSEKVQANVKNMLLLEESN GYKIFGKTGWA MDI         KPQVGWLTGWVEQ
OXA-24     PLKITPVQEVNFADDLAHNRLPFKLETQEEVKKMLLIKEVN GSKIYAKSGWG MGV         TPQVGWLTGWVEQ
OXA-45     SLKISPEGQVRFVRDLLSAKLPASKDAQQMTVSILPHFAAG DWAVQGKTGTG SFIDAR   GAKAPLGWFIGWATH
OXA-46     NLKISAHEQILFLRKLYRNQLPFKVEHQRLVKDLMITEAGR SWILRAKTGWE G--        --RFGWWVGWIEW
OXA-48     GIRISATEQISFLRKLYHNKLHVSERSQRIVKQAMLTEANG DYIIRAKTGYS TRI         EPKIGWWVGWVEL
OXA-51     PLKITPQQEAQFAYKLANKTLPFSPKVQDEVQSMLFIEEKN GNKIYAKSGWG WDV         DPQVGWLTGWVVQ
OXA-54     GIRISATEQVAFLRKLYHNKLHVSERSQRIVKQAMLTEANS DYIIRAKTGYS TRI         EPQIGWWVGWVEL
OXA-58     PLTITPIQEVKFVYDLAQGQLPFKPEVQQQVKEMLYVERRG ENRLYAKSGWG MAV         DPQVGWYVGFVEK
OXA-66     PLKITPQQEAQFAYKLANKTLPFSQKVQDEVQSMLFIEEKN GNKIYAKSGWG WDV         NPQVGWLTGWVVQ
OXA-85     PLKISAMEQVKLLNLLSQSKLPFKLENQEQVKDITILEKKD DFILHGKTGWA TDN        IVVPIGWFVGWIET
OXA-143    PLKITPIQEVNFADDFANNRLPFKLETQEEVKKMLLIKEFN GSKIYAKSGWG MDV         TPQVGWLTGWVEK
OXA-145    QLRISAVNQVEFLESLFLNKLSASKENQLIVKEALVTEAAP EYLVHSKTGFS GVGTE      SNPGVAWWVGWVEK
OXA-146    PLKVTPIQEVEFVSQLAHTQLPFSEKVQANVKNMLLLEESN GYKIFGKTGWAAMDI         KPQVGWLTGWVEQ
OXA-160    PLKITPVQEVNFADDLAHNRLPFKLETQEEVKKMLLIKEVN GSKIYAKSGWG MGV         TSQVGWLTGWVEQ
OXA-163    GIRISATEQISFLRKLYHNKLHVSERSQRIVKQAMLTEANG DYIIRAKTGYD T--         --KIGWWVGWVEL
OXA-181    GIRISATQQIAFLRKLYHNKLHVSERSQRIVKQAMLTEANG DYIIRAKTGYS TRI         EPKIGWWVGWVEL
OXA-225    PLKVTPIQEVEFVSQLAHTQLPFSEKVQANVKNMLLLEESN GYKIFGKTGWA MDI         KSQVGWLTGWVEQ
OXA-231    PLKITPIQEVNFADDFANNRLPFKLETQEEVKKMLLIKEFN GSKIYAKSGWG MAV         TPQVGWLTGWVEK
OXA-232    GIRISATQQIAFLRKLYHNKLHVSERSQRIVKQAMLTEANG DYIIRAKTGYS TSI         EPKIGWWVGWVEL
OXA-239    PLKVTPIQEVEFVSQLAHTQLPFSEKVQANVKNMLLLEESN GYKIFGKTGWA MNI         KSQVGWLTGWVEQ
OXA-245    GIRISATEQISFLRKLYHNKLHVSERSQRIVKQAMLTEANG DYIIRAKTGYS TRI         EPKIGWWVGWVEL
OXA-405    GIRISATEQISFLRKLYHNKLHVSERSQRIVKQAMLTEANG DYIIRAKTGYS P--         --KIGWWVGWVEL
OXA-427    SLAISPQEQARFLGKLVSGKLPVSAQTLQHTANILRQPDID GWQIHGKTGTG YPKLLDGSLDRDQQIGWFVGWASK
OXA-436    GIRISATQQIAFLRKLYHNKLHVSERSQRIVKQAMLTEANA DYIIRAKTGYS VRI         EPKIGWWVGWIEL
OXA-517    GIRISATEQISFLRKLYHNKLHVSERSQRIVKQAMLTEANG DYIIRAKTGYS T--         KPKIGWWVGWVEL
OXA-655    QLRISAVNQVEFLESLYLNKLSASKENQLIVKEALVTEAAP EYLVHSKTGFS GVGTE      SNPGVAWWVGWVEK
OXA-935    QLRISAVNQVEFLESLYLNKLSASKENQLIVKEALVTEAAP EYLVHSKTGFS GVGTE      SNPGVAWWVGWVEK
OXA-D84    SLQISPLEQLRFLSKMLNRKLPVSPNAVDMTERIVEATTLADGTVVHGKTGAA YPLLADGTRDWAHGFGWFVGWITR
AFD-1      SLTISPVEQVGFLRRLLGGNLPFSRDAQAKTRAIMPVFDAPESWAVHGKTGTG YMRDEKGNPDRNRPFGWFVGWAER
ATD-1      SLTVSPVEQVGFIRRLLAGNLPVSRDAQAKTRAIVPVFDAPESWSVHGKTGTG FMRDEKGNPDRSRPFGWFVGWAER
CPD-1      SLQISPDEELGFVKKLYFDQLPFHKVTMQNVRQVMLMEKKP EYELSYKTGMG FSG         PKTIGWITGWIEE
Lox-A9     SLSISPTEQIQFLQKIIYKKLPVSQKAYTMTKNIMYIQELPGGWKLYGKTGTG RQLTKDKSQKLPLQHGWFVGWIEK
NOD-1      PLQITPKQQIEFLQRLHRKELPFSQRTLDLVQDIMIYERTP NYVLRGKTGWA ASV         TPNIGWFVGYLEQ
STD-1      SLKISPEEQIDFLINLYEEKFMLSEKTYKIVKDIMINEKTP EYTLRGKTGWG REG         AENIIWYVGYIEA
                                                -201a     -215a -215b -215c -215d -215e -215f -215g -215h

consensus                                              KTG                    GWxxGW
SAND #                 170       180       190       200       210                    220
```

```
              β7                      α11
OXA-1     KSGHKYVFVSALTGNLGS NLTSSIKAKKNAITILNTLNL
OXA-2     PT GSVFFALNIDTPNRM   DDLFKREAIVRAILRSIEALPPNPAVNSDAAR
OXA-10    ET EVYFFAFNMDIDNE    SKLPLRKSIPTKIMESEGIIGG
OXA-13    GT EVYFFAFNMDIDNE    NKLPLRKSIPTKIMASEGIIGG
OXA-14    ET EVYFFAFNMDIDNE    SKLPLRKSIPTKIMESEGIIGG
OXA-17    ET EVYFFAFNMDIDNE    SKLPLRKSIPTKIMESEGIIGG
OXA-23    PDGKIVAFALNMEMRSE    MPASIRNELLMKSLKQLNII
OXA-24    ANGKKIPFSLNLEMKEG    MSGSIRNEITYKSLENLGII
OXA-45    EE RRVVFARMTAGGKKG EQPAGPAARDAFLKALPDLAKRF
OXA-46    PT GPVFFALNIDTPNRT   DDLFKREAIARAILRSIDALPPN
OXA-48    DD NVWFFAMNMDMPTS    DGLGLRQAITKEVLKQEKIIP
OXA-51    PQGNIVAFSLNLEMKKG    IPSSVRKEITYKSLEQLGIL
OXA-54    DD NVWFFAMNMDMPTA    DGLGLRQAITKEVLKQEKIIP
OXA-58    ADGQVVAFALNMQMKAG    DDIALRKQLSLDVLDKLGVFHYL
OXA-66    PQGNIVAFSLNLEMKKG    IPSSVRKEITYKSLEQLGIL
OXA-85    SD NIYSFAINLDISDS    KFLPKREEIVREYFKNINVIK
OXA-143   SNGEKVAFSLNIEMKQG    MPGSIRNEITYKSLENLGII
OXA-145   GT EVYFFAFNMDIDNE    NKLPLRKSIPTKIMASEGIIGG
OXA-146   PDGKIVAFALNMEMRSE    MPASIRNELLMKSLKQLNII
OXA-160   ANGKKIPFSLNLEMKEG    MSGSIRNEITYKSLENLGII
OXA-163   DD NVWFFAMNMDMPTS    DGLGLRQAITKEVLKQEKIIP
OXA-181   DD NVWFFAMNMDMPTS    DGLGLRQAITKEVLKQEKIIP
OXA-225   PDGKIVAFALNMEMRSE    MPASIRNELLMKSLKQLNII
OXA-231   SNGEKVAFSLNIEMKQG    MPGSIRNEITYKSLENLGII
OXA-232   DD NVWFFAMNMDMPTS    DGLGLRQAITKEVLKQEKIIP
OXA-239   PDGKIVAFALNMEMRSE    MPASIRNELLMKSLKQLNII
OXA-245   DD NVWFFAMNMDMPTS    DGLGLRQAITKEVLKQEKIIP
OXA-405   DD NVWFFAMNMDMPTS    DGLGLRQAITKEVLKQEKIIP
OXA-427   QD QKLIFVHTVIQKPGKQ FASLRAREEVFAALPEQLKKL
OXA-436   DD NVWFFATNMDMPTA    EGLGLRQTITKAVLKQEKIIP
OXA-517   DD NVWFFAMNMDMPTS    DGLGLRQAITKEVLKQEKIIP
OXA-655   ET EVYFFAFNMDIDNE    SKLPLRKSIPTKIMESEGIIGG
OXA-935   ET EVYFFAFNMDIDNE    SKLPLRKSIPTKIMESEGIIGG
OXA-D84   GN QTLVFARLMQDERKQ PVSTGIRTREAFLRDLPRLLATR
AFD-1     EG QHIVFARLRVSDKPS NEPLGPAVRDAFLRDIPRLAVHR
ATD-1     EG QHIVFARLRVADKPS SEPLGPAVRDAFLRDIARLAVHR
CPD-1     NG HPSFFVLNIETENKS  LDMRTVRMNILRNLLTDAGYFKGMK
Lox-A9    DE RVITFAKHIADSKEN TTFASFRAKNDTLIQLFNLINELEK
NOD-1     NN NVYFFATNIDIRNN    DDAAARIEVTRRSLKALGLL
STD-1     KE NVYFFAVRIINASEERNSYLLDFRKQLTMMAFRELGIIN
             -230a          -244a -244b -244c -244d
consensus
SAND #    230       240          250       260       270
```

Figure S2 (this page and the three preceding pages). Structure-based sequence alignment of Gram-negative DBLs. The 7 sequence motifs are colored blue (*motif-1*), pink (*motif-2*), green (*motif-3*), magenta (*motif-4*), orange (*motif-5*), cyan (*motif-6*) and yellow (*motif-7*). The P-loop, Ω-loop and B-loop are highlighted in gray. The SAND numbering scheme (based on OXA-48 residue numbering) is given at the bottom of each block. In this scheme any residues missing relative to OXA-48 are skipped in numbering and shown as a dash (-) to maintain alignment. Where there is an insertion in an aligned sequence relative to OXA-48, lower case letters are added to the residue number that just precedes the insertion, and these numbers are shown vertically on the alignment

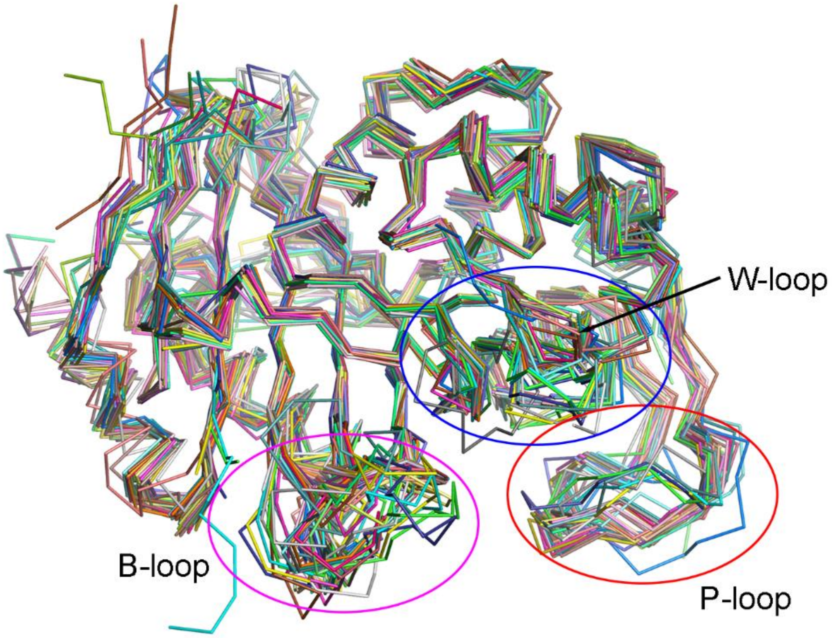

Figure S3. Structural superposition of the Gram-negative DBLs. The P-loop, Ω-loop and B-loop are indicated.

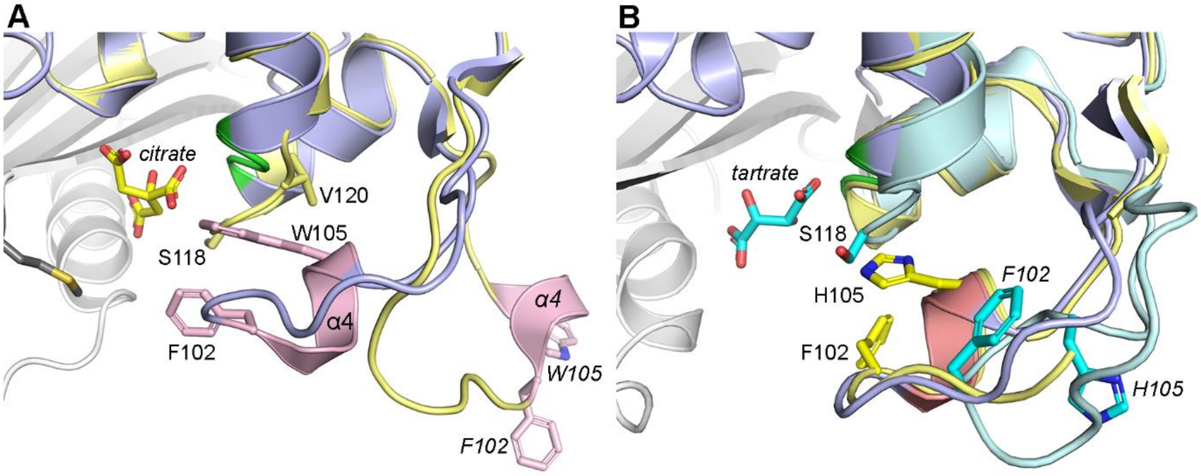

Figure S4. DBL P-loop flexibility. **(A)** Two crystal forms of OXA-23 at neutral pH (light blue and gray ribbons) and at acidic pH (yellow ribbons). The alternate conformation of the α5-α6 loop in the low pH form (indicated by Ser118 and Val120 in yellow) would overlap with the Trp105 sidechain, hence the rearrangement of the low pH P-loop. The citrate anion is shown as yellow sticks. **(B)** Alternate conformations of the P-loop in OXA-45 monomer B (pale cyan ribbons and cyan sticks) and monomer C (yellow ribbons and sticks), superimposed on the type-I P-loop from OXA-48 (light blue and gray ribbons). The tartrate anion in monomer B is shown as cyan sticks.

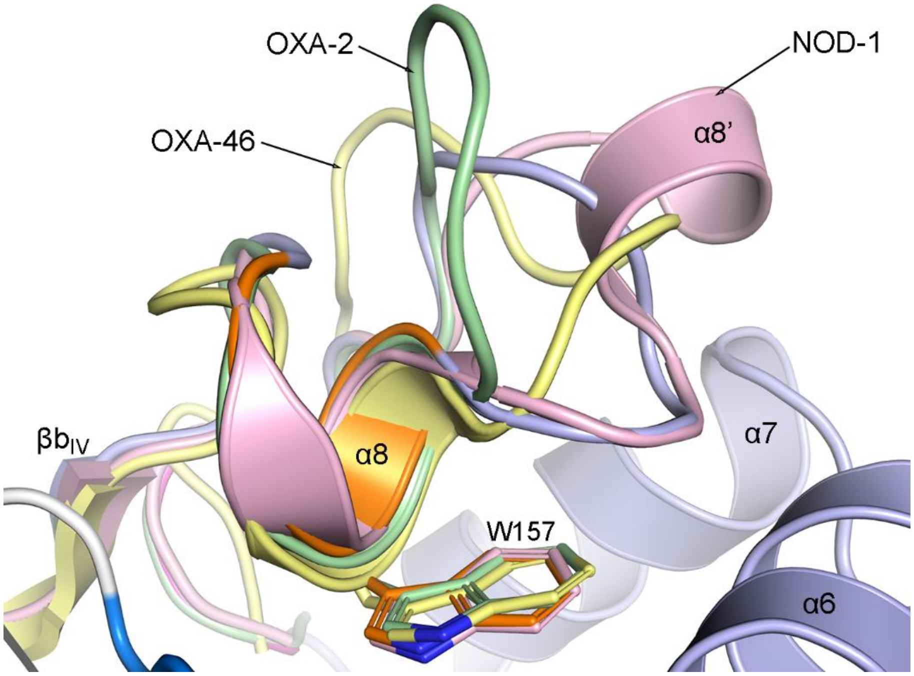

Figure S5. Atypical Ω-loops in the GnDBLs. The loops for OXA-2 (green), OXA-46 (yellow) and NOD-1 (pink) are superimposed onto the type-I loop in OXA-48 (light blue). Importantly, the Trp157 residue is isostructural irrespective of the loop structure.

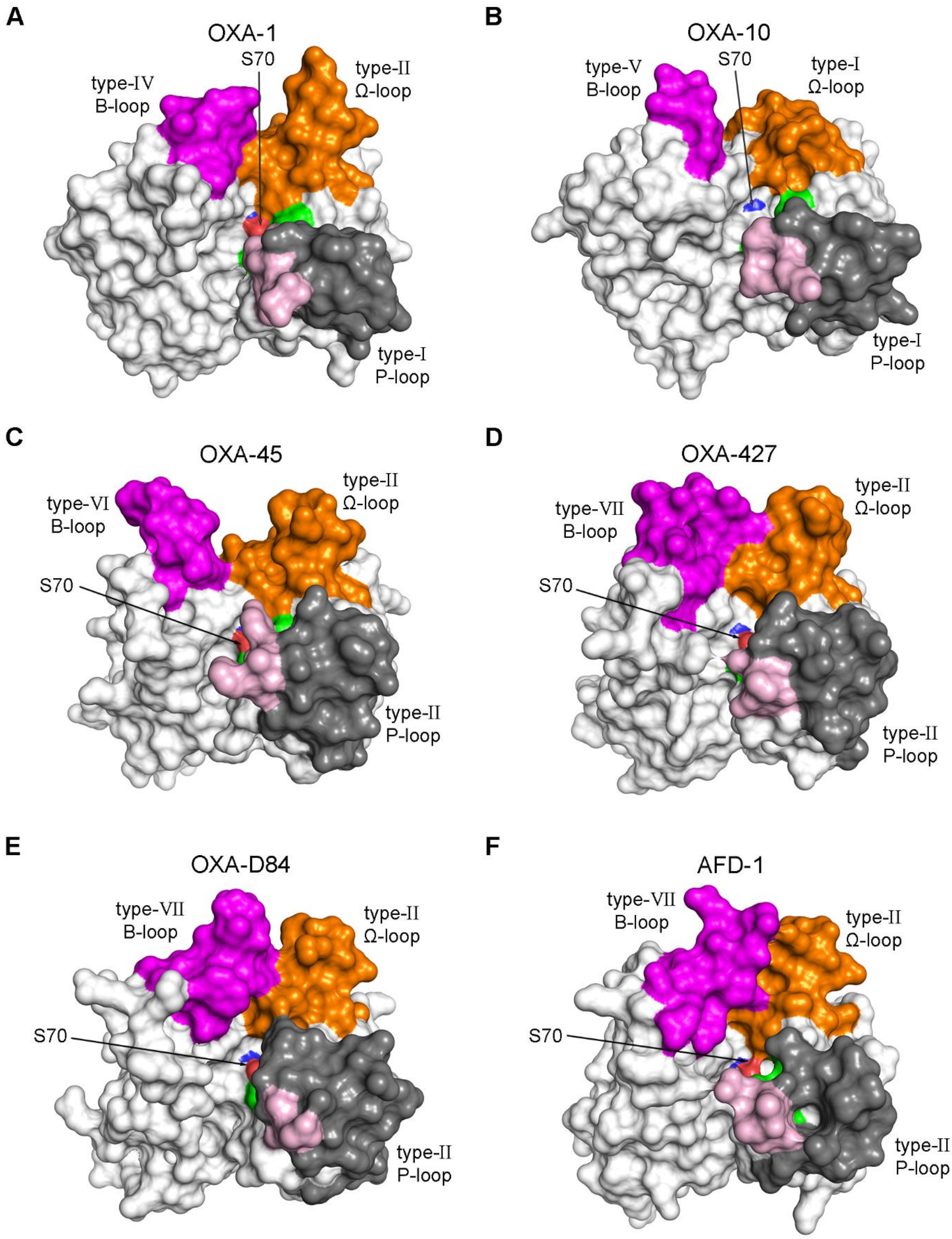

Figure S6. Molecular surface representations of several GnDBLs. **(A)** OXA-1. **(B)** OXA-10 **(C)** OXA-45. **(D)** OXA-427. **(E)** OXA-D84. **(F)** AFD-1.

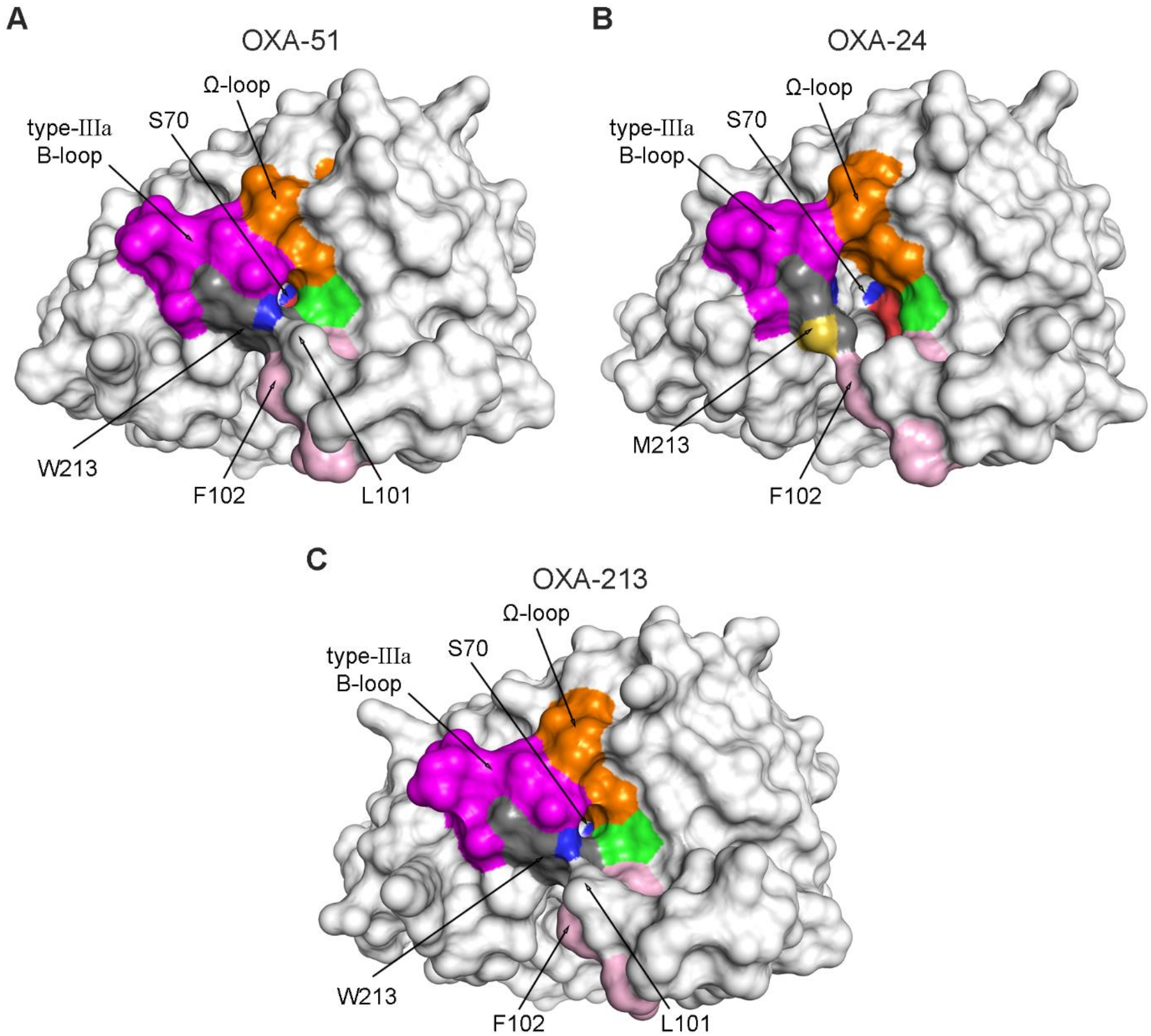

Figure S7. Molecular surfaces representations. **(A)** OXA-51, showing the expansive hydrophobic bring formed between Trp213, Leu101 and Phe102. **(B)** OXA-24, with a smaller hydrophobic bridge formed by Met213 and Phe102. **(C)** The AlphaFold2 model of OXA-213, again with a large hydrophobic bridge.

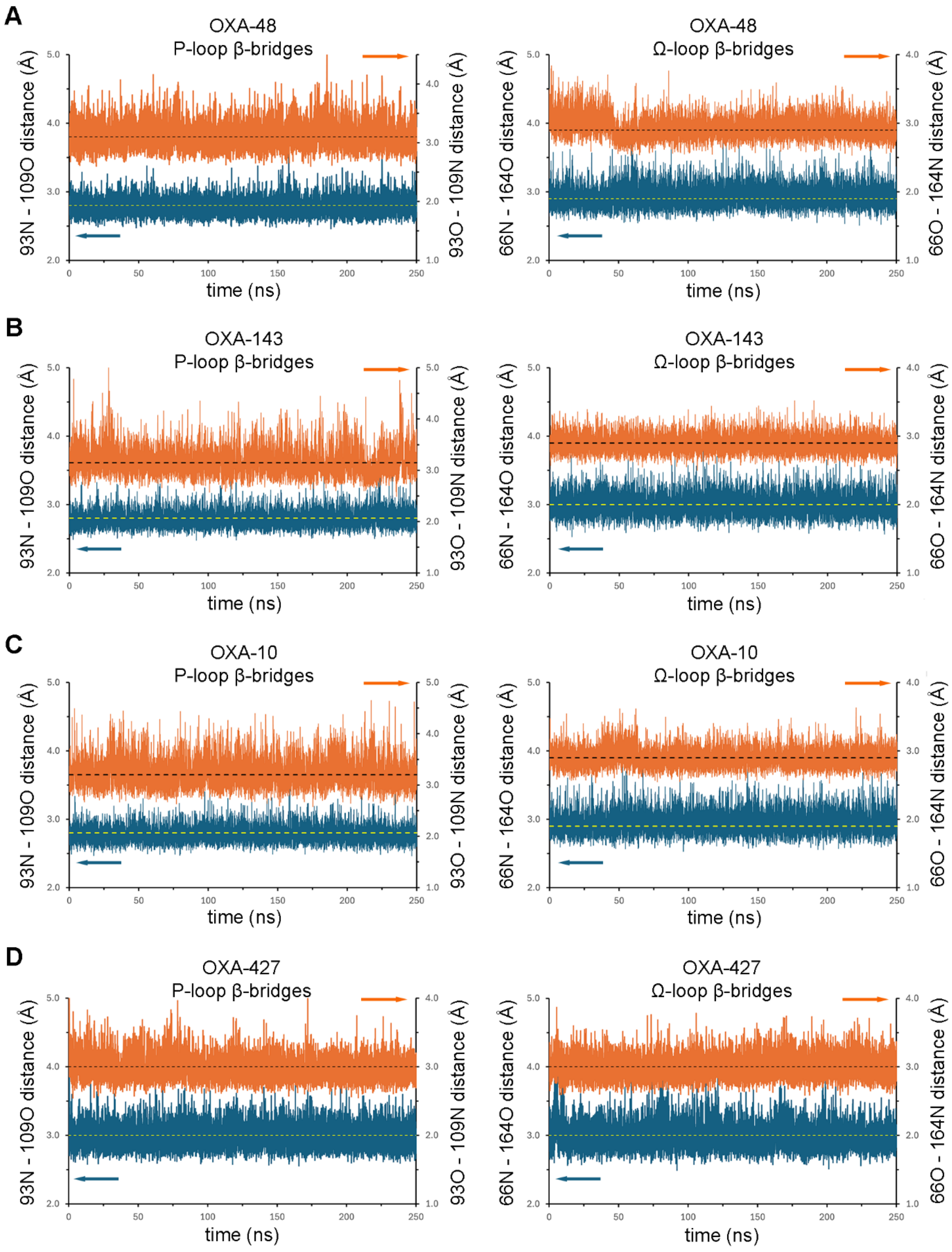

Figure S8. Hydrogen bonding in the β-bridges. The left vertical axes relate to the blue traces, and the right vertical axes refer to the orange traces. Average values for the blue and orange traces are shown as yellow and black dashed lines, respectively. **(A)** OXA-48; average distances are 3.1 Å and 2.8 Å (blue and orange traces) for the P-loop

bridges, and 2.9 Å (both blue and orange traces) for the Ω-loop. **(B)** OXA-143; average distances are 2.8 Å and 3.2 Å (blue and orange traces) for the P-loop bridges, and 3.0 Å and 2.9 Å (blue and orange traces) for the Ω-loop. **(C)** OXA-10; average distances are 2.8 Å and 3.2 Å (blue and orange traces) for the P-loop bridges, and 2.9 Å (both blue and orange traces) for the Ω-loop. **(D)** OXA-427; average distances are 3.0 Å (both blue and orange traces) for the P-loop bridges, and 3.0 Å (both blue and orange traces) for the Ω-loop.

Supplementary Tables

Table S1. GnDBL enzyme families[a]

OXA with > 10 members	OXA with 5-10 members	OXA with < 5 members	Other families
OXA-1 (19)	OXA-22 (7)	OXA-5 (3)	AFD-1/ATD-1 (2)
OXA-2 (33)	OXA-46 (6)	OXA-9 (2)	LCR-1/NPS-1 (2)
OXA-10 (69)	OXA-55 (9)*	OXA-20 (2)	RSD2 (2)
OXA-12 (11)	OXA-58 (10)	OXA-42 (4)	
OXA-23 (54)*	OXA-60 (7)*	OXA-62 (3)*	
OXA-24 (15)*	OXA-214 (7)*	OXA-198 (4)*	
OXA-48 (69)*	OXA-243 (10)	OXA-258 (3)*	
OXA-50 (117)	OXA-274 (6)*	OXA-266 (2)	
OXA-51 (398)*	OXA-548 (6)	OXA-279 (3)	
OXA-61 (49)	OXA-679 (7)*	OXA-308 (3)	
OXA-63 (23)	OXA-1258 (6)	OXA-372 (3)*	
OXA-114 (23)		OXA-427 (2)*	
OXA-134 (31)*		OXA-464 (4)	
OXA-143 (13)*		OXA-493 (2)	
OXA-184 (39)		OXA-664 (4)	
OXA-211 (18)*		OXA-732 (2)	
OXA-213[b] (123)*		OXA-1043 (2)	
OXA-229 (27)*		OXA-1149 (4)*	
OXA-286 (17)*			
OXA-294 (13)*			
OXA-504 (12)			

[a] A family is determined to be a grouping of two or more sequences with greater than 90% identity. Those marked with an asterisk have been annotated as carbapenemases, and the families colored red are from *Acinetobacter* sp.

[b] Includes the OXA-270 sub-family (85-90% identity to OXA-213)

Table S2. GnDBL structures

Enzyme	Total structures	Family	PDB code	Resolution (Å)	Space group	Unit cell (a, b, c; Å)	P-loop type	Ω-loop type	β5-β6 type	Reference
AFD-1	1	AFD/ATD	6NHU	2.3	$P2_1$	49.88, 165.96, 75.43, β = 90.0°	II	II	VIIa	NP
ATD-1	1	AFD/ATD	6V6N	1.85	$P2_1$	58.12, 101.66, 108.14, β = 97.8°	II	II	VIIa	NP
CPD-1	2	-	7K3M	1.8	$P2_12_12_1$	49.42, 69.35, 70.04	I	I	IIIc	NP
LoxA-9	1	-	6WHL	2.3	$P2_1$	80.22, 178.20, 143.75, β = 97.04	II	II	VIIa	NP
NOD-1	1	-	6NHS	2	$P6_222$	116.50, 116.50, 74.70	I	atypical	IIIa	NP
OXA-1	3	1	4MLL	1.37	P1	50.92, 72.61, 73.45,<br>α = 80.9°, β = 69.9°, γ = 71.4°	I	II	IV	[2]
OXA-2	2	2	6XJ3	1.85	$P2_1$	69.55. 88.67, 76.10, β = 99.2°	I	atypical	I	NP
OXA-10	44	10	1E3U	1.66	$P2_1$	65.53, 82.94, 101.42	I	I	V	[3] [4]
OXA-13	3	10	1H8Y	2.0	$P2_12_12_1$	45.34, 112.30, 124.98	I	I	V	[5]
OXA-14	3	10	7L5R	1.65	$P2_12_12_1$	48.81, 96.35, 125.14	I	I	V	[6]
OXA-17	1	10	9IXQ	1.98	$P2_12_12_1$	48.56 102.81 126.75	I	I	V	[7]
OXA-23	15	23	4JF4	2.14	$P2_1$	49.47, 44.53, 131.37	I	I	IIIa	[8]
OXA-24	30	24	2JC7	2.5	$P4_12_12$	102.20, 102.20, 86.10	I	I	IIIa	[9]
OXA-45	1	-	4GN2	2.01	$P2_12_12_1$	45.17, 47.489, 108.90	II	II	VI	NP
OXA-46	1	46	3IF6	2.4	H32	123.84, 123.84, 327.92	I	atypical	I	[10]
OXA-48	91	48	3HBR	1.9	$P2_1$	63.70, 107.18, 80.78	I	I	IIIa	[11]
OXA-51	3	51	4ZDX	2	I422	131.11, 131.11, 67.53	I	I	IIIa	[12]
OXA-54	1	48	6NLW	1.85	C2	174.33, 61.39, 122.80, β = 119.5°	I	I	IIIa	NP
OXA-57[a]	1	42	9HPT	1.8	$C222_1$	65.79, 110.35, 64.80	-[a]	-[a]	-	[13]
OXA-58	9	58	4OH0	1.3	$P2_12_12_1$	37.12, 65.41, 93.76	I	I	IIIa	[14]

OXA-66	1	51	6T1H	2.1	$P4_322$	87.54, 87.54, 90.12	I	I	IIIa	NP
OXA-85	1	-	4IED	1.5	P1	43.95, 65.67, 91.69<br>α = 84.6°, β = 82.9°, γ = 70.1°	I	I	IIIe	NP
OXA-143	1	143	5IY2	1.15	$P2_1$	40.96, 62.33, 87.09, β = 91.7	I	I	IIIa	[15]
OXA-145	1	10	4YIN	2.3	$P2_12_12_1$	46.73, 91.18, 125.28	I	I	V	[16]
OXA-146	1	23	4K0W	1.2	$P2_122_1$	44.12, 46.66, 137.02	I	I	IIIb	[17]
OXA-160	2	24	4X53	2.3	$P4_12_12$	102.45, 102.45, 87.22	I	I	IIIc	[18]
OXA-163	6	48	4S2L	1.72	$P2_1$	44.87, 125.75, 49.71, β = 116.8°	I	I	I	[19]
OXA-181	1	48	5OE0	2.05	$P6_2$	143.93, 143.93, 53.54	I	I	IIIa	[20]
OXA-225	1	23	4X55	1.94	$P2_12_12$	98.78, 143.86, 44.18	I	I	IIIc	[18]
OXA-231	1	143	6NZ8	1.2	$P2_12_12_1$	39.79, 54.79, 96.24	I	I	IIIa	[21]
OXA-232	1	48	5HFO	2.21	$P6_2$	144.08, 144.08, 53.13	I	I	IIIa	[22]
OXA-239	3	23	5WI3	1.81	$P2_12_12$	98.10, 143.03, 44.31	I	I	IIId	[23]
OXA-245	1	48	5OE2	2.2	$P2_1$	64.16, 108.72, 83.68, β = 102.4°	I	I	IIIa	[20]
OXA-405	1	48	5FDH	2.26	$P4_32_12$	90.40, 90.40, 172.63	I	I	I	[24]
OXA-427	1	427	6HUH	2.78	C2	143.79, 42.58, 99.71, β = 114.4°	II	II	VIIb	NP
OXA-436	1	48	7ODA	1.8	$P2_1$	69.12, 94.57, 87.76, β = 109.9°	I	I	IIIa	[25]
OXA-517	1	48	6HB8	1.86	$P2_1$	49.80, 125.045, 82.66, β = 94.7°	I	I	II	[26]
OXA-655	1	10	6SKQ	2.1	$P2_1$	68.14, 82.44, 99.00, β = 97.0°	I	I	V	[27]
OXA-935	2	10	7L5V	1.3	$P2_1$	46.07, 75.04, 82.75, β = 91.9°	I	disordered	V	[6]
OXA-D84	1	42	6NI0	2.3	$P4_12_12$	43.20, 43.20, 256.90	II	II	VIIa	NP
STD-1	1	NF	6N1N	1.6	$P2_12_12_1$	63.51, 97.55, 40.33	I	I	IIIc	NP

[a] This structure has not yet been released in the PDB but has been published as a preprint.

References

1. Naas, T.; Oueslati, S.; Bonnin, R.A.; Dabos, M.L.; Zavala, A.; Dortet, L.; Retailleau, P.; Iorga, B.I. Beta-Lactamase DataBase (BLDB) – Structure and Function. *J. Enzyme Inhib. Med. Chem.* **2017**, *32*, 917-919. doi: 10.1080/14756366.2017.1344235
2. June, C.M.; Vallier, B.C.; Bonomo, R.A.; Leonard, D.A.; Powers, R.A. Structural Origins of Oxacillinase Specificity in Class D β-Lactamases. *Antimicrob. Agents Chemother.* **2014**, *58*, 333-341. doi: 10.1128/AAC.01483-13
3. Maveyraud, L.; Golemi, D.; Kotra, L.P.; Tranier, S.; Vakulenko, S.; Mobashery, S.; Samama, J.P. Insights Into Class D β-Lactamases are Revealed by the Crystal Structure of the OXA10 Enzyme from *Pseudomonas Aeruginosa*. *Structure* **2000**, *8*, 1289-1298. doi: 10.1016/S0969-2126(00)00534-7
4. Paetzel, M.; Danel, F.; de Castro, L.; Mosimann, S.C.; Page, M.G.P.; Strynadka, N.C.J. Crystal structure of the class D β-lactamase OXA-10. *Nature Struct. Biol.* **2000**, *7*, 918-924. doi: 10.1038/79688
5. Pernot, L.; Frénois, F.; Rybkine, T.; L'Hermite, G.; Petrella, S.; Delettré, J.; Jarlier, V.; Collatz, E.; Sougakoff, W. Crystal structures of the class D β-lactamase OXA-13 in the native form and in complex with meropenem. *J. Mol. Biol.* **2001**, *310*, 859-874. doi: 10.1006/jmbi.2001.4805
6. Pincus, N.B.; Rosas-Lemus, M.; Gatesy, S.W.M.; Bertucci, H.K.; Brunzelle, J.S.; Minasov, G.; Shuvalova, L.A.; Lebrun-Corbin, M.; Satchell, K.J.F.; Ozer, E.A.; et al. Functional and Structural Characterization of OXA-935, a Novel OXA-10-Family β-Lactamase from *Pseudomonas aeruginosa*. *Antimicrob. Agents Chemother.* **2022**, *66*, e0098522. doi: 10.1128/aac.00985-22
7. Lee, C.; Park, Y.; Park, H.; Kwak, K.; Lee, H.; Yun, J.; Lee, D.; Lee, J.H.; Lee, S.H.; Kang, L.-W. Structural insights into alterations in the substrate spectrum of serine-β-lactamase OXA-10 from *Pseudomonas aeruginosa* by single amino acid substitutions. *Emerg. Microbes Infect.* **2024**, *13*, 2412631. doi: 10.1080/22221751.2024.2412631
8. Smith, C.A.; Antunes, N.T.; Stewart, N.K.; Toth, M.; Kumarasiri, M.; Chang, M.; Mobashery, S.; Vakulenko, S.B. Structural basis for carbapenemase activity of the OXA-23 β-lactamase from *Acinetobacter baumannii*. *Chem. Biol.* **2013**, *20*, 1107-1115. doi: 10.1016/j.chembiol.2013.07.015

9. Santillana, E.; Beceiro, A.; Bou, G.; Romero, A. Crystal structure of the carbapenemase OXA-24 reveals insights into the mechanism of carbapenem hydrolysis. *Proc. Natl. Acad. Sci.* **2007**, *104*, 5354-5359. doi: 10.1073/pnas.0607557104

10. Docquier, J.D.; Benvenuti, M.; Calderone, V.; Giuliani, F.; Kapetis, D.; De Luca, F.; Rossolini, G.M.; Mangani, S. Crystal structure of the narrow-spectrum OXA-46 class D β-lactamase: relationship between active-site lysine carbamylation and inhibition by polycarboxylates. *Antimicrob. Agents Chemother.* **2010**, *54*, 2167-2174. doi: 10.1128/AAC.01517-09

11. Docquier, J.D.; Calderone, V.; De Luca, F.; Benvenuti, M.; Giuliani, F.; Bellucci, L.; Tafi, A.; Nordmann, P.; Botta, M.; Rossolini, G.M.; et al. Crystal structure of the OXA-48 β-lactamase reveals mechanistic diversity among class D carbapenemases. *Chem. Biol.* **2009**, *16*, 540-547. doi: 10.1016/j.chembiol.2009.04.010

12. Smith, C.A.; Antunes, N.T.; Stewart, N.K.; Frase, H.; Toth, M.; Kantardjieff, K.A.; Vakulenko, S. Structural Basis for Enhancement of Carbapenemase Activity in the OXA-51 Family of Class D β-Lactamases. *ACS Chem. Biol.* **2015**, *10*, 1791-1796. doi: 10.1021/acschembio.5b00090

13. Bragginton, E.C.; Colenso, C.K.; Calvopiña, K.; Hinchliffe, P.; Shaw, J.M.; Tooke, C.L.; Seng, R.; Chantratita, N.; Mulholland, A.J.; Schofield, C.J.; et al. Structure and dynamics of Burkholderia pseudomallei OXA-57, a distinctive low efficiency class D β-lactamase with carbapenem-hydrolyzing activity. *bioRxiv* **2024**, 2024.2012.2023.630153, doi:doi.org/10.1101/2024.12.23.630153.

14. Smith, C.A.; Antunes, N.T.; Toth, M.; Vakulenko, S.B. Crystal Structure of Carbapenemase OXA-58 from *Acinetobacter baumannii*. *Antimicrob. Agents Chemother.* **2014**, *58*, 2135–2143. doi: 10.1128/AAC.01983-13

15. Toth, M.; Smith, C.A.; Antunes, N.T.; Stewart, N.K.; Maltz, L.; Vakulenko, S.B. The role of conserved surface hydrophobic residues in the carbapenemase activity of the class D β-lactamases. *Acta Crystallogr.* **2017**, *D73*, 692-701. doi: 10.1107/S2059798317008671

16. Meziane-Cherif, D.; Bonnet, R.; Haouz, A.; Courvalin, P. Structural insights into the loss of penicillinase and the gain of ceftazidimase activities by OXA-145 β-lactamase in *Pseudomonas aeruginosa*. *J. Antimicrob. Chemother.* **2016**, *71*, 395-402. doi: 10.1093/jac/dkv375

17. Kaitany, K.C.; Klinger, N.V.; June, C.M.; Ramey, M.E.; Bonomo, R.A.; Powers, R.A.; Leonard, D.A. Structures of the class D carbapenemases OXA-23 and OXA-146: Mechanistic basis of activity against carbapenems, extended-spectrum cephalosporins and aztreonam. *Antimicrob. Agents Chemother.* **2013**, *58*, 4848-4855. doi: 10.1128/AAC.00762-13
18. Mitchell, J.M.; Clasman, J.R.; June, C.M.; Kaitany, K.C.; LaFleur, J.R.; Taracila, M.A.; Klinger, N.V.; Bonomo, R.A.; Wymore, T.; Szarecka, A.; et al. Structural Basis of Activity against Aztreonam and Extended Spectrum Cephalosporins for Two Carbapenem-Hydrolyzing Class D β-Lactamases from *Acinetobacter baumannii*. *Biochemistry* **2015**, *54*, 1976-1987. doi: 10.1021/bi501547k
19. Stojanoski, V.; Chow, D.C.; Fryszczyn, B.; Hu, L.; Nordmann, P.; Poirel, L.; Sankaran, B.; Prasad, B.V.; Palzkill, T. Structural Basis for Different Substrate Profiles of Two Closely Related Class D β-Lactamases and Their Inhibition by Halogens. *Biochemistry* **2015**, *54*, 3370-3380. doi: 10.1021/acs.biochem.5b00298
20. Lund, B.A.; Thomassen, A.M.; Carlsen, T.J.O.; Leiros, H.K.S. Structure, activity and thermostability investigations of OXA-163, OXA-181 and OXA-245 using biochemical analysis, crystal structures and differential scanning calorimetry analysis. *Acta Crystallogr.* **2017**, *F73*, 579-587. doi: 10.1107/S2053230X17013838
21. Antunes, V.U.; Llontop, E.E.; da Costa Vasconcelos, F.N.; Lopez de los Santos, Y.; Oliveira, R.J.; Lincopan, N.; Farah, C.S.; Doucet, N.; Mittermaier, A.; Favaro, D.C. Importance of the β5-β6 Loop for the Structure, Catalytic Efficiency, and Stability of Carbapenem-Hydrolyzing Class D β-Lactamase Subfamily OXA-143. *Biochemistry* **2019**, *58*, 3604-3616. doi: 10.1021/acs.biochem.9b00365
22. Oueslati, S.; Retailleau, P.; Marchini, L.; Berthault, C.; Dortet, L.; Bonnin, R.A.; Iorga, B.I.; Naas, T. Role of Arginine 214 in the Substrate Specificity of OXA-48. *Antimicrob. Agents Chemother.* **2020**, *64*, 10.1128/aac.02329-02319. doi: 10.1128/AAC.02329-19
23. Harper, T.M.; June, C.M.; Taracila, M.A.; Bonomo, R.A.; Powers, R.A.; Leonard, D.A. Multiple substitutions lead to increased loop flexibility and expanded specificity in *Acinetobacter baumannii* carbapenemase OXA-239. *Biochem. J.* **2018**, *475*, 273-288. doi: 10.1042/BCJ20170702

24. Oueslati, S.; Retailleau, P.; Marchini, L.; Dortet, L.; Bonnin, R.A.; Iorga, B.I.; Naas, T. Biochemical and Structural Characterization of OXA-405, an OXA-48 Variant with Extended-Spectrum β-Lactamase Activity. *Microorganisms* **2020**, *8*, 24. doi: 10.3390/microorganisms8010024
25. Lund, B.A.; Thomassen, A.M.; Carlsen, T.J.W.; Leiros, H.-K.S. Biochemical and biophysical characterization of the OXA-48-like carbapenemase OXA-436. *Acta Crystallogr.* **2021**, *F77*, 312-318. doi: 10.1107/S2053230X21008645
26. Dabos, L.; Raczynska, J.E.; Bogaerts, P.; Zavala, A.; Girlich, D.; Bonnin, R.A.; Dortet, L.; Peyrat, A.; Retailleau, P.; Iorga, B.I.; et al. Structural and Biochemical Features of OXA-517: A Carbapenem and Expanded-Spectrum Cephalosporin Hydrolyzing OXA-48 Variant. . *Antimicrob. Agents Chemother.* **2023**, *67*, e0109522. doi: 10.1128/aac.01095-22
27. Leiros, H.-K.S.; Thomassen, A.M.; Samuelsen, O.; Flach, C.-F.; Kotsakis, S.D.; Larsson, D.G.J. Structural insights into the enhanced carbapenemase efficiency of OXA-655 compared to OXA-10. *FEBS Open Bio* **2020**, *10*, 1821-1832. doi: 10.1002/2211-5463.12935
